# Atomic Resolution Description of the Interaction between the Nucleoprotein and Phosphoprotein of Hendra Virus

**DOI:** 10.1371/journal.ppat.1003631

**Published:** 2013-09-26

**Authors:** Guillaume Communie, Johnny Habchi, Filip Yabukarski, David Blocquel, Robert Schneider, Nicolas Tarbouriech, Nicolas Papageorgiou, Rob W. H. Ruigrok, Marc Jamin, Malene Ringkjøbing Jensen, Sonia Longhi, Martin Blackledge

**Affiliations:** 1 Université Grenoble Alpes, Institut de Biologie Structurale (IBS), Grenoble, France; 2 CEA, DSV, IBS, Grenoble, France; 3 CNRS, IBS, Grenoble, France; 4 Université Grenoble Alpes, UVHCI, Grenoble, France; 5 CNRS, UVHCI, Grenoble, France; 6 Unit for Virus Host Cell Interactions, Université Grenoble Alpes-EMBL-CNRS, Grenoble, France; 7 CNRS and Aix-Marseille Université, Architecture et Fonction des Macromolécules Biologiques, UMR 7257, Marseille, France; Institut Pasteur, France

## Abstract

Hendra virus (HeV) is a recently emerged severe human pathogen that belongs to the *Henipavirus* genus within the *Paramyxoviridae* family. The HeV genome is encapsidated by the nucleoprotein (N) within a helical nucleocapsid. Recruitment of the viral polymerase onto the nucleocapsid template relies on the interaction between the C-terminal domain, N_TAIL_, of N and the C-terminal X domain, XD, of the polymerase co-factor phosphoprotein (P). Here, we provide an atomic resolution description of the intrinsically disordered N_TAIL_ domain in its isolated state and in intact nucleocapsids using nuclear magnetic resonance (NMR) spectroscopy. Using electron microscopy, we show that HeV nucleocapsids form herringbone-like structures typical of paramyxoviruses. We also report the crystal structure of XD of P that consists of a three-helix bundle. We study the interaction between N_TAIL_ and XD using NMR titration experiments and provide a detailed mapping of the reciprocal binding sites. We show that the interaction is accompanied by α-helical folding of the molecular recognition element of N_TAIL_ upon binding to a hydrophobic patch on the surface of XD. Finally, using solution NMR, we investigate the interaction between intact nucleocapsids and XD. Our results indicate that monomeric XD binds to N_TAIL_ without triggering an additional unwinding of the nucleocapsid template. The present results provide a structural description at the atomic level of the protein-protein interactions required for transcription and replication of HeV, and the first direct observation of the interaction between the X domain of P and intact nucleocapsids in *Paramyxoviridae*.

## Author Summary

The polymerase of negative strand RNA viruses reads the viral RNA that is associated with the nucleoprotein N forming a helical nucleocapsid. The interaction between N and the cofactor of the polymerase, the phosphoprotein P, is essential for transcription and replication of the viral genome. The mechanism by which the polymerase dislodges the RNA from the nucleoprotein for its polymerising activity remains unknown, although it has been proposed that binding to P causes a conformational change in the nucleocapsid. Here, we use nuclear magnetic resonance (NMR) spectroscopy to develop an atomic resolution description of the intrinsically disordered C-terminal domain, N_TAIL_, of N of Hendra virus, an emerging paramyxovirus, and X-ray crystallography to determine the structure of the X domain (XD) of P. Characterization of the interaction between XD and N_TAIL_ provides evidence for folding of N_TAIL_ upon binding to P. Crucially, we were also able to study, for the first time, the interaction between XD and recombinant paramyxoviral nucleocapsids. NMR spectra of N_TAIL_ in its isolated form and in the context of nucleocapsids demonstrate that binding of XD does not change the dynamics of N_TAIL_ and that the nucleocapsid does not undergo any major rearrangements or unwinding upon interaction with P.

## Introduction

The Hendra (HeV) and Nipah (NiV) viruses are recently emerged, severe human pathogens within the *Paramyxoviridae* family [1]. A few distinctive properties set them aside from other paramyxoviruses and led to their classification within the *Henipavirus* genus of the *Paramyxoviridae* family that also contains the newly identified Cedar virus [2], [3]. The genome of Henipaviruses is encapsidated by the nucleoprotein (N) within a helical nucleocapsid. This helical N∶RNA complex, rather than the naked RNA, serves as substrate for both transcription and replication [4]. By analogy with other paramyxoviruses, the viral polymerase complex is thought to consist of the L protein and the phosphoprotein P. The P protein is an essential polymerase cofactor as it tethers L onto the nucleocapsid template [5], [6]. *Henipavirus* N and P proteins were shown to interact with each other [7]–[9], being able to form both homologous and heterologous N-P complexes [9]–[11]. The functional significance of the N-P interaction for genome transcription and replication makes it a promising target for antiviral drug design [12].

So far, high-resolution structural data are limited to *Henipavirus* surface proteins, where crystallographic studies led to the determination of the three-dimensional structure of the fusion (F) and the attachment (G) proteins [13], [14]. A more complete picture of the molecular mechanisms governing transcription and replication of the virus awaits an atomic resolution characterization of the three key players N, P and L. The only available molecular data on *Henipavirus* N and P proteins come from our previous studies [7]–[9], [15] showing that the N and P proteins possess a modular organization consisting of both ordered and intrinsically disordered regions [15], [16]. Intrinsically disordered proteins (IDPs) are ubiquitous, functional proteins that lack significant amounts of secondary and tertiary structure under physiological conditions and are, therefore, not amenable to characterization by X-ray crystallography [17]–[21]. IDPs populate a vast conformational space and ensemble descriptions have emerged as the preferred tool for representing their highly dynamic nature. In this context, nuclear magnetic resonance (NMR) spectroscopy, in combination with ensemble selection techniques, has contributed immensely to our understanding of the conformational behaviour of IDPs providing a detailed mapping of their inherent potential energy landscape [22]–[29].

**Figure 1:**
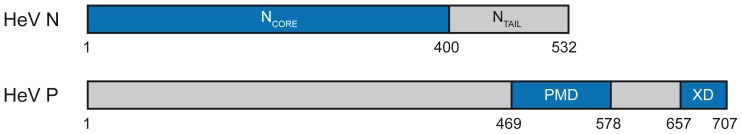
Domain organization of HeV N and P proteins. Gray areas indicate regions of the two proteins that are predicted to be intrinsically disordered.

The HeV nucleoprotein (N) consists of two domains: a folded domain (referred to as N_CORE_, residues 1–400) that is responsible for the interaction with the viral RNA and for maintaining the nucleocapsid structure, and an intrinsically disordered domain (referred to as N_TAIL_, residues 401–532) that is responsible for the interaction with the P protein (Figure 1) [7]–[10]. The atomic resolution structure of N_CORE_ remains unknown, although, the crystal structure of the decameric ring-like N∶RNA complex of a related paramyxovirus, the respiratory syncytial virus (RSV), has been solved recently [30]. The structure reveals two domains of the nucleoprotein, where the RNA is accommodated in a positively charged pocket at the hinge between the two domains [31].

The HeV phosphoprotein (P) consists of a large intrinsically disordered N-terminal domain (residues 1–469), a central domain (PMD, residues 470–578) that is thought to be responsible for the oligomerization of P through a coiled-coil arrangement by analogy with the related Sendai (SeV) [32] and Measles viruses (MeV) [33], and a flexible linker of 80 amino acids connecting the globular C-terminal X domain (XD, residues 657–707) to PMD (Figure 1) [15].

In this study, we provide an atomic resolution description of the structure and dynamics of both N_TAIL_ and XD using a combination of nuclear magnetic resonance (NMR) spectroscopy and X-ray crystallography. The complex between N_TAIL_ and XD is characterised using NMR revealing a characteristic folding of the molecular recognition element (MoRE) of N_TAIL_ upon binding to XD. In addition, we determine the conformational behaviour of N_TAIL_ in the context of intact nucleocapsids revealing that N_TAIL_ remains flexible, as in the case of MeV nucleocapsids, and exhibits differential flexibility along its primary sequence. While previous solution NMR studies investigated the residual structure and dynamics of N_TAIL_ in the context of intact MeV nucleocapsids, we here describe the first study of the interaction between a paramyxoviral nucleocapsid and the X domain of the phosphoprotein. The present results reveal that HeV XD can be accommodated on N_TAIL_ without triggering additional unwinding of the helical nucleocapsid template. Our results provide an atomic resolution description of the protein-protein interactions that play an essential role in transcription and replication of HeV.

## Results

### Residual structure and dynamics of HeV N_TAIL_

**Figure 2:**
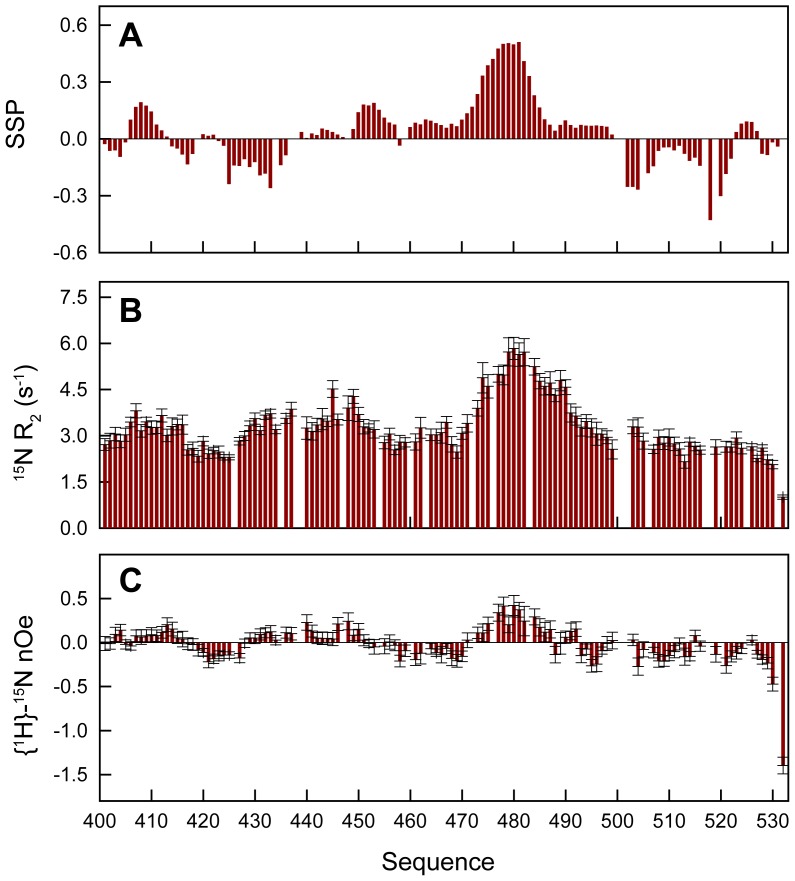
Characterization of the structure and dynamics of HeV N_TAIL_. (A) Secondary structure propensity (SSP) of N_TAIL_ obtained from experimental Cα and Cβ chemical shifts. (B) ^15^N *R*_2_ relaxation rates of N_TAIL_ at 293 K and a ^1^H frequency of 600 MHz. (C) {^1^H}-^15^N steady state heteronuclear Overhauser effects of N_TAIL_ at 293 K and a ^1^H frequency of 600 MHz.

In order to characterize the residual structure and dynamics of N_TAIL_, we carried out the spectral assignment of the backbone and Cβ nuclei using standard triple resonance experiments (Figure S1, Table S1 in Text S1). Carbon chemical shifts are exquisitely sensitive to the presence of secondary structure allowing us to directly probe the nature and level of residual secondary structure within N_TAIL_. The secondary structure propensity (SSP) of N_TAIL_ was calculated on the basis of the experimental Cα and Cβ chemical shifts (Figure 2A) [34]. Positive and negative SSP scores indicate propensity to form α-helical and β-extended conformations, respectively. The SSP shows that, although N_TAIL_ is devoid of significant secondary structure, the protein adopts an α-helical conformation in the region encompassing residues 470–490. The helical propensity reaches approximately 50% for the central residues and decreases progressively towards the extremities. This region of the protein has previously been shown to constitute the binding site of the P protein [7]–[9].

^15^N *R*_2_ relaxation rates and {^1^H}-^15^N heteronuclear Overhauser effects (nOe) report on dynamics occurring on the pico- to nanosecond time scale and were used to probe the backbone dynamics of N_TAIL_. They display a profile characteristic of an IDP revealing, however, more elevated values in the region 470–490 due to a reduced level of chain flexibility arising from the residual helical structure present in this region (Figure 2B,C) [35]. In addition, the glycine and serine-rich regions 417–425, 455–470 and the C-terminal part display on average higher flexibility on the pico- to nanosecond time scale compared to the remainder of the chain.

### Three-dimensional structure of HeV XD

**Figure 3:**
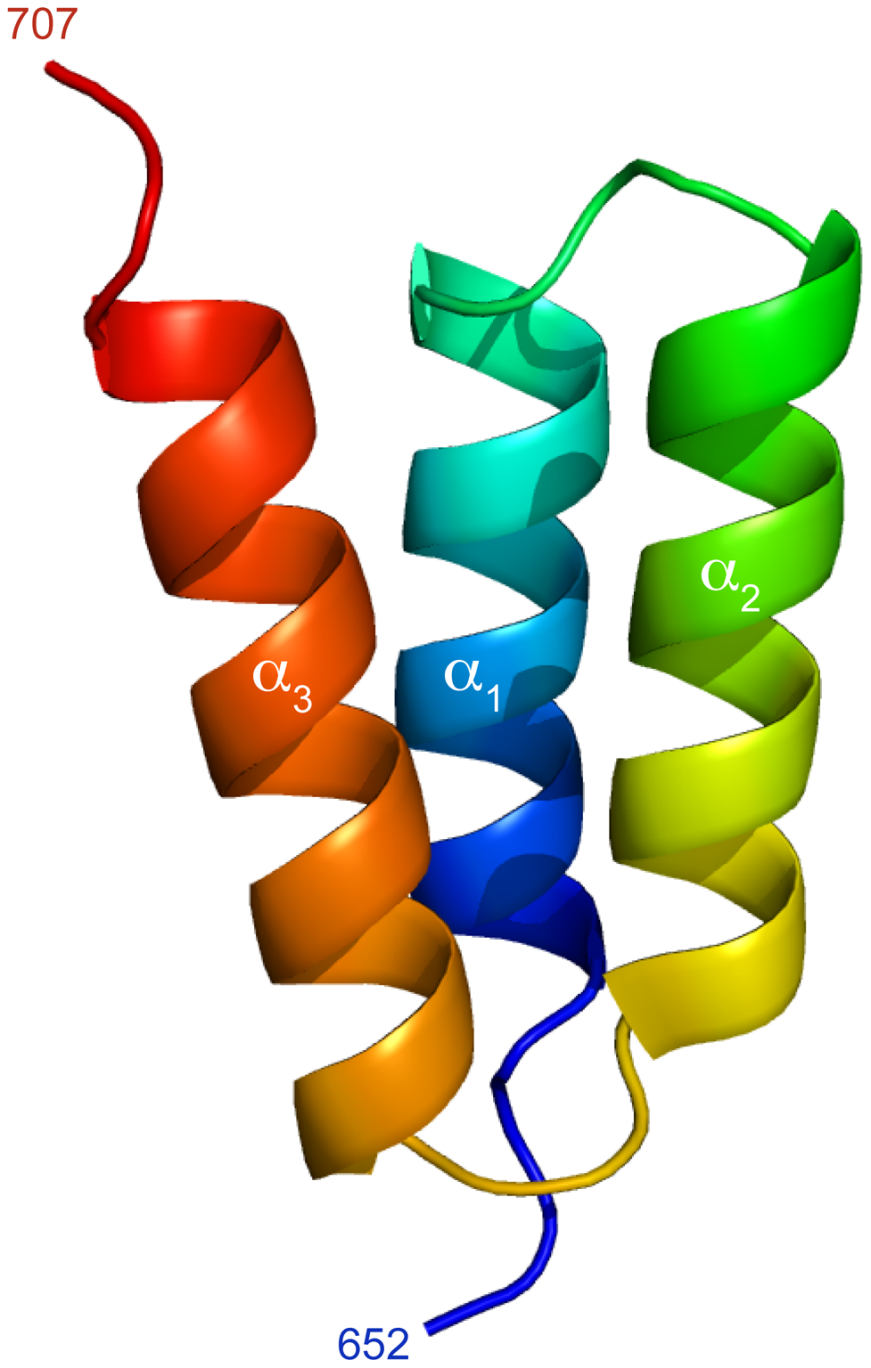
Crystal structure of HeV XD. Cartoon representation of the structure of XD in the crystal. The model is coloured from blue at the N-terminus to red at the C-terminus.

The three-dimensional crystal structure of HeV XD was determined from X-ray diffraction data. The protein crystallized in a P2_1_ space group with two molecules within the asymmetric unit of the crystal. Crystallographic phases were determined by single wavelength anomalous diffraction (SAD) using synchrotron data collected from crystals of the selenomethionine-substituted protein, and the structure was refined at 1.65 Å resolution (Table S2 in Text S1). The electron density was well defined throughout the structure, except for a few side chains on the surface, and the model of chain B includes four additional residues encoded by the expression vector (LEHH). The structure is composed of three α-helices, forming an anti-parallel three-helix bundle (Figure 3). The two copies in the asymmetric unit are equivalent (RMSD = 0.65 Å) and interact through a molecular surface (900 Å^2^) created by helices α1 and α3.

**Figure 4:**
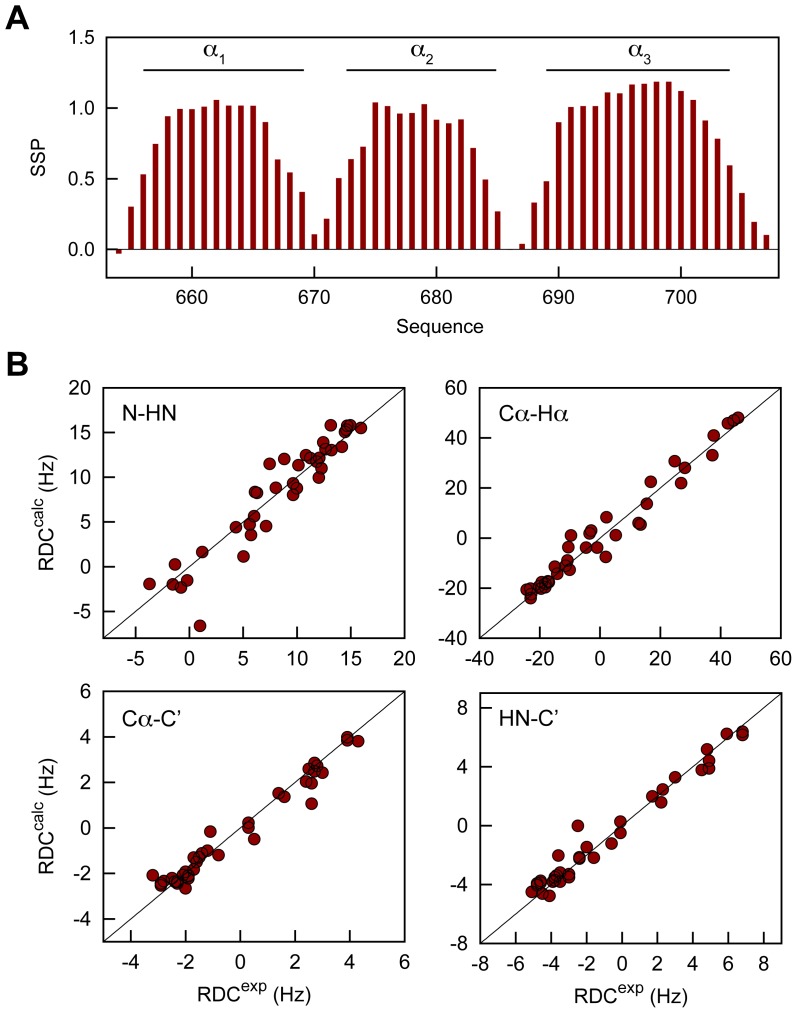
Structure of HeV XD in solution. (A) Secondary structure propensity (SSP) of XD obtained from experimental Cα and Cβ chemical shifts. (B) Agreement between experimental residual dipolar couplings obtained of XD in a suspension of filamentous phages and those back-calculated on the basis of the crystal structure of XD.

To investigate whether the structure of XD in solution is identical to the one determined by crystallography, as well as to study the interaction of XD with N_TAIL_, we carried out the NMR backbone assignment of XD on the basis of a set of standard triple resonance experiments (Figure S2, Table S3 in Text S1). The location of secondary structure elements obtained from the experimental Cα and Cβ chemical shifts are consistent with a three-helix bundle arrangement (Figure 4A). We also measured NMR residual dipolar couplings (RDCs) by weakly aligning the protein molecule in the magnetic field [36]–[38]. RDCs report on bond vector orientations with respect to a global alignment tensor that describes the preferential orientation of the molecule in the magnetic field. Four types of RDCs measured in a concentrated suspension of filamentous phages agree well with those back-calculated from the structure determined by X-ray crystallography (Table S4 in Text S1, Figure 4B). The RDCs, therefore, confirm that the structure adopted by XD in solution is very similar to that observed in the crystal.

### Atomic level structural insights into the interaction between HeV N_TAIL_ and XD

**Figure 5:**
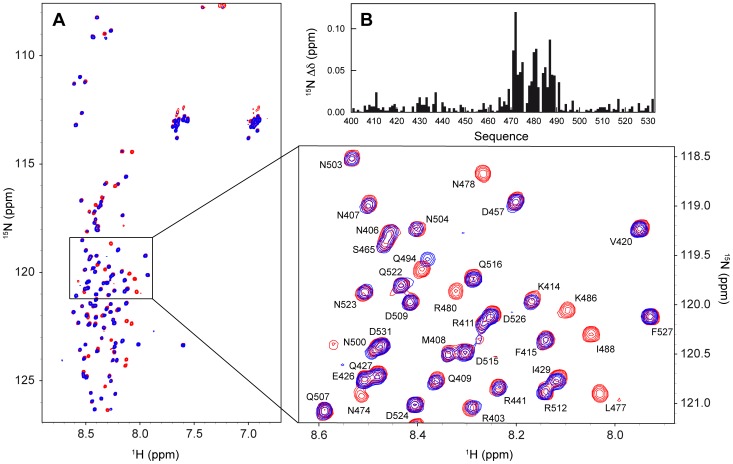
Interaction between ^15^N labeled N_TAIL_ and unlabeled XD. (A) Superposition of the ^1^H-^15^N HSQC spectra of isolated N_TAIL_ (red) and N_TAIL_ containing 4.4-fold molar excess of XD (blue). The spectra were obtained at a ^1^H frequency of 800 MHz and 293 K. The expanded region shows the spectral assignment of a number of resonances in the spectrum of the isolated N_TAIL_ domain. (B) ^15^N chemical shift changes (absolute values) between isolated N_TAIL_ and N_TAIL_ with 30% of XD (molar ratio) added. Note that at this molar fraction all the peaks of N_TAIL_ are still visible.

To map the reciprocal N_TAIL_ and XD interaction sites, as well as to gain insight into the molecular mechanisms controlling the binding reaction, NMR chemical shift titration experiments were performed. The addition of an increasing amount of unlabeled XD to ^15^N labeled N_TAIL_ results in severe line broadening of the NMR resonances for residues 470–490 even at sub-stoichiometric amounts of XD (Figure 5A). This behaviour is a manifestation of contributions to the transverse relaxation rates arising from exchange occurring on the micro- to millisecond time scale between free and bound conformations of N_TAIL_. This confirms that the residues 470–490 are the primary interaction site of N_TAIL_ with XD. The largest chemical shift perturbations in N_TAIL_ are also observed in the region of residues 470–490 (Figure 5B, Figure S3 in Text S1). The affinity of the interaction between N_TAIL_ and XD could not be derived from the chemical shift titrations as the resonances of the residues of N_TAIL_ that are involved in the interaction disappear when more than 30% (molar fraction) of XD is added to N_TAIL_. The effective dissociation constant was previously measured by isothermal titration calorimetry to be 8.7 µM [7].

**Figure 6:**
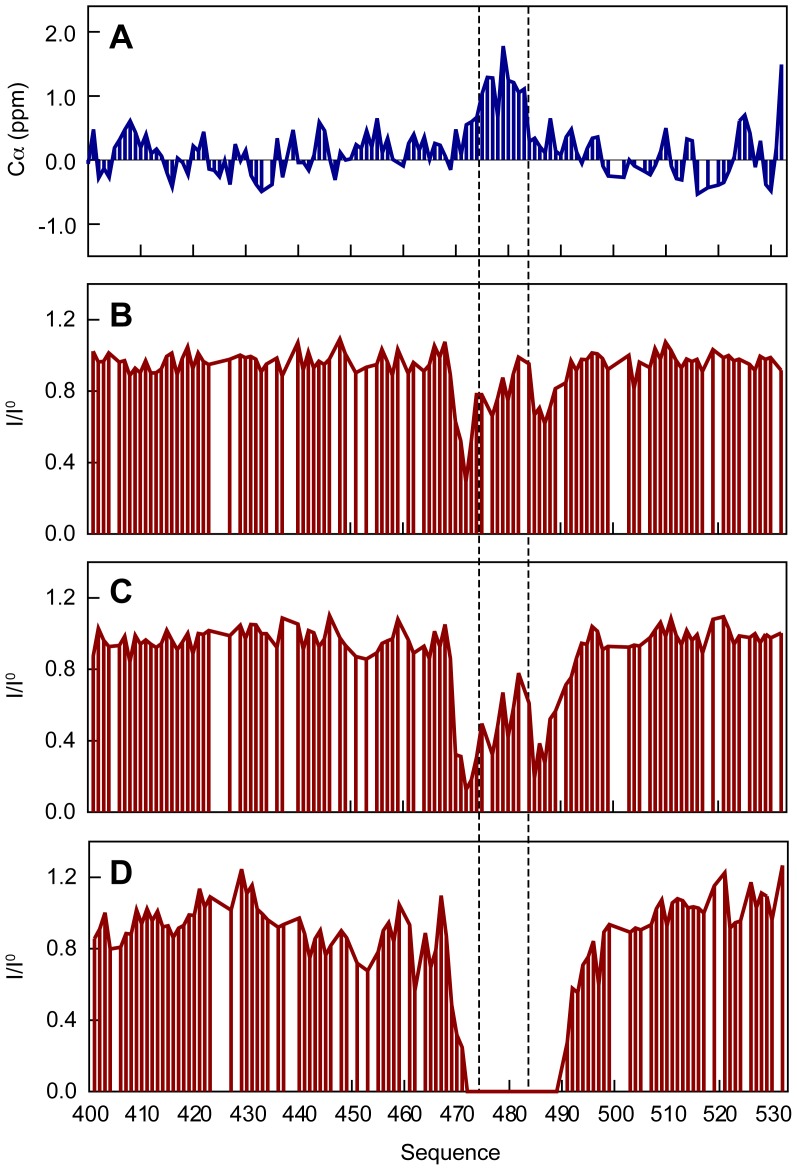
Conformational exchange contributions to the NMR line widths of N_TAIL_ upon interaction with XD. (A) Secondary Cα chemical shifts of N_TAIL_. (B)–(D) Intensity profiles of the ^1^H-^15^N HSQC spectra of N_TAIL_ with different amounts of XD: 7.5% (B), 30% (C), 440% (D). The profiles are calculated as *I*/*I* ^0^, where *I* are the intensities in the spectrum of N_TAIL_ with XD, and *I* ^0^ are the intensities in the spectrum of the isolated N_TAIL_ domain. All intensity profiles were normalized to one outside the MoRE. In all panels the dashed lines indicate the region for which most helical propensity is present in the isolated state of N_TAIL_ according to the secondary Cα chemical shifts.

To obtain further mechanistic details of the interaction between N_TAIL_ and XD, we monitored the decrease in signal intensities in the ^1^H-^15^N HSQC spectra of N_TAIL_ at each XD titration point (Figure 6B, C, D). Interestingly, the signal intensity decreases faster for the residues located at the extremities of the MoRE and for which a smaller amount of residual helical structure is observed experimentally in the isolated state of N_TAIL_ (Figure 6A). This differential broadening suggests that N_TAIL_ binds to XD via a short, central helix, which is subsequently extended via helical folding of the adjacent residues. Our data therefore indicate that N_TAIL_ interacts with XD via a folding-upon-binding mechanism [39]–[41]. The line broadening observed in the interaction site implies that the folding event occurs on the micro- to millisecond time scale.

**Figure 7:**
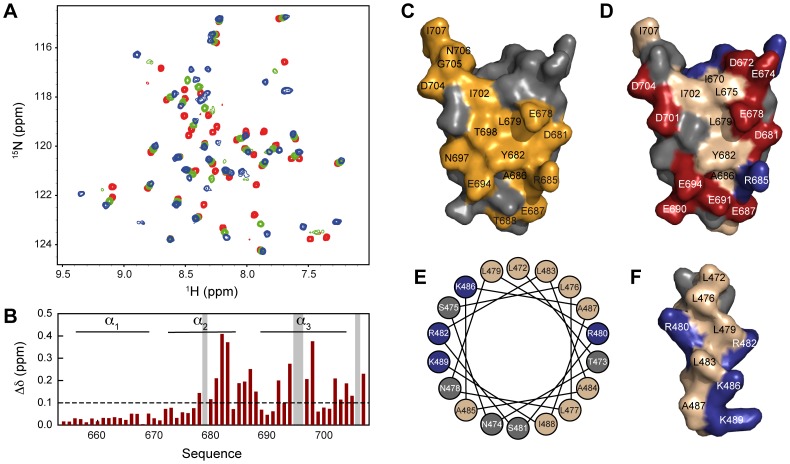
Interaction between ^15^N, ^13^C labeled XD and unlabeled N_TAIL_. (A) Superposition of ^1^H-^15^N HSQC spectra of XD containing increasing amounts of N_TAIL_: 0% (red), 71% (green) and 416% (blue). The spectra were obtained at a ^1^H frequency of 600 MHz and 298 K. (B) Combined ^1^H and ^15^N chemical shift difference (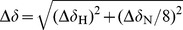) between the resonances in the ^1^H-^15^N HSQC spectrum of isolated XD and the spectrum with 416% N_TAIL_. Gray vertical shading indicates residues for which the NMR resonances disappear upon addition of N_TAIL_. Chemical shift changes above the horizontal dashed line are considered significant. (C) Surface representation of the crystal structure of XD displaying residues experiencing large chemical shift changes upon addition of N_TAIL_ (orange). The orientation of XD is the same as shown in Figure 3. (D) Surface representation of the crystal structure of XD displaying the location of hydrophobic (beige), negatively charged (red) and positively charged (blue) residues. (E) Helical wheel representation of the MoRE of N_TAIL_ encompassing residues 472–489 with the same color-coding as in (D). (F) Surface representation of the MoRE of N_TAIL_ assuming that the MoRE adopts an α-helical conformation.

In order to map the interaction site of N_TAIL_ on XD, we carried out the complementary titration, monitoring chemical shift changes in ^15^N, ^13^C labeled XD upon addition of unlabeled N_TAIL_. The resonances of XD shift and gradually lose intensity with increasing N_TAIL_ concentration, and then regain intensity when approaching saturation (Figure 7A). This suggests that the exchange between the free and bound form of XD occurs on a fast to intermediate timescale (faster than the millisecond) in agreement with the complementary titration of N_TAIL_. The largest chemical shift and intensity changes are observed for residues of helix α2 and α3 identifying these two helices as the site of interaction for N_TAIL_ (Figure 7B, C). Only small chemical shift changes are observed in helix α1.

The crystal structure of XD provides additional information about the mechanism of interaction between N_TAIL_ and XD. The distribution of hydrophobic and charged residues on the surface of XD reveals a hydrophobic groove between helix α2 and α3 surrounded by numerous negatively charged residues (Figure 7D). We propose that the negatively charged residues on the surface of XD play a role in correctly orienting N_TAIL_ before adopting its final conformation in the hydrophobic pocket [42]. This is supported by the distribution of hydrophobic and charged residues within the MoRE of N_TAIL_. Assuming a helical conformation of the bound MoRE [7], [8], a helical wheel representation of this region shows four leucines (L472, L476, L479 and L483) and an alanine (A487) clustered on one side of the N_TAIL_ molecular recognition helix most likely constituting the direct interaction interface with XD (Figure 7E, F). In addition, four positively charged residues (R480, R482, K486 and K489) flank the four leucines and the alanine providing charge complementarity with respect to the negatively charged residues on the surface of XD. Note that this orientation of the MoRE at the XD surface is consistent with previous electron paramagnetic resonance (EPR) data showing that the extent to which the N_TAIL_ side chains are constrained within the N_TAIL_-XD complex follows the order A487>S481>S475 [8].

### Characterization of N_TAIL_ in the context of intact HeV nucleocapsids

**Figure 8:**
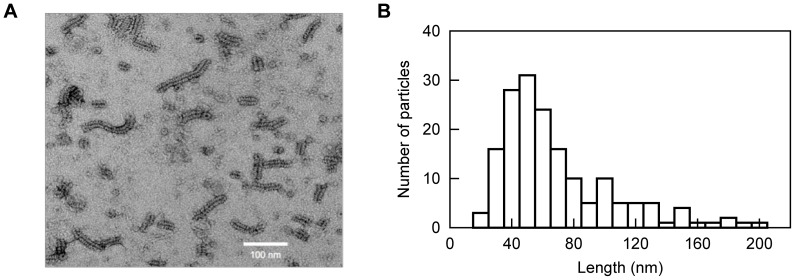
Electron microscopy of intact HeV nucleocapsids. (A) Negative stain electron micrograph of HeV N showing the presence of nucleocapsid-like structures. (B) Histogram showing the distribution of lengths of 170 randomly chosen nucleocapsids.

**Figure 9:**
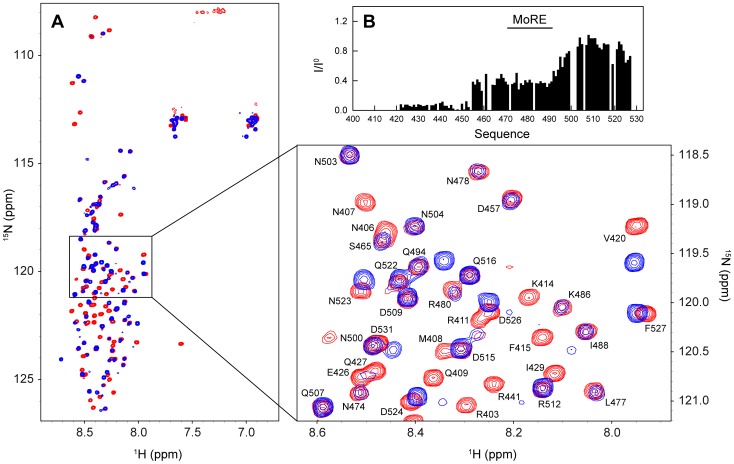
Characterization of N_TAIL_ in the context of intact nucleocapsids. (A) Superposition of the ^1^H-^15^N HSQC spectra of isolated N_TAIL_ (red) and intact nucleocapsids (blue). The spectra were obtained at a ^1^H frequency of 600 MHz and 293 K. The expanded region shows the spectral assignment of a number of resonances in the spectrum of the isolated N_TAIL_ domain. (B) Normalized intensity profile of the HeV nucleocapsid ^1^H-^15^N HSQC spectrum. The profile is calculated as *I*/*I* ^0^, where *I* are the intensities in the spectrum of the nucleocapsids and *I* ^0^ are the intensities in the spectrum of the isolated N_TAIL_ domain.

In order to study the conformational behaviour of N_TAIL_ in the context of the full-length nucleoprotein, we expressed and purified ^15^N labeled, C-terminally His-tagged N (see Materials and Methods). Expression of full-length N results in the formation of nucleocapsid-like particles containing bacterial RNA (the nucleocapsid-like particles are simply denoted nucleocapsids in the following). In order to image the structures formed by HeV N, we performed negative staining transmission electron microscopy studies. As shown in Figure 8A, the sample contains flexible nucleocapsids with the typical herringbone appearance previously reported for other paramyxoviruses [43]–[50]. Figure 8B shows the nucleocapsid length statistics as inferred from analysis of 170 different particles. The analysis shows that the nucleocapsids have lengths between 20 and 200 nm with more than 75% of the nucleocapsids in the range between 30 and 90 nm. Assuming a mean pitch of 5.7 nm as determined previously for MeV [51], the majority of the nucleocapsids contain 5 to 15 turns. The large size of the nucleocapsids (where 5–15 turns with 12 protomers per turn yields a mass in the order of 2.5 to 24 MDa) would normally preclude the detection of solution NMR signals due to the absence of fast rotational tumbling. However, the spectrum of the intact HeV nucleocapsids displays a large number of resonances and superimposes well on that of the isolated N_TAIL_ domain with only minor chemical shift changes (Figure 9A). This shows that N_TAIL_ retains significant conformational flexibility *in situ* and allows the resonance assignment of the isolated N_TAIL_ domain to be transferred to the nucleocapsid spectrum. Only the five C-terminal residues (528–532) of N_TAIL_ could not be easily identified in the nucleocapsid spectrum due to the different location of the His-tag in the two proteins.

The intensity profile of the nucleocapsid spectrum (normalized with respect to the spectrum of the isolated N_TAIL_ domain) reveals that N_TAIL_ has differential flexibility along its primary sequence (Figure 9B). The resonances of residues 401–454 of N_TAIL_ are absent or have very low signal intensities indicating that N_TAIL_ is too rigid in this part of the sequence to give rise to solution NMR signals. This observation closely mimics measurements made previously on recombinant nucleocapsids of MeV [52] and indicates that the N_TAIL_ domain of HeV also requires approximately 50 residues to exfiltrate from the spindle to the exterior of the nucleocapsid helix. By assuming that each turn of the helical nucleocapsid contains 12 nucleoproteins and a pitch of 5.7 nm as determined previously for MeV [51], we estimate on the basis of our length distribution (Figure 8B) that 9% of the N_TAIL_ domains detected by NMR are located at the edge of the nucleocapsids. This percentage is very close to the experimental intensity ratio observed for residues 420–450, suggesting that the residual intensities in this region are nucleocapsid-edge effects that arise from N_TAIL_ domains that are not positioned between two helical turns of the nucleocapsid (Figure 9B). These intensities can therefore be considered as an artefact of the purification of recombinant nucleocapsids.

The NMR signal intensities of the region encompassing residues 455–490 of N_TAIL_ increase to an approximately constant level, while the C-terminal residues 491–527 gain even further in signal intensity (Figure 9B). The plateau observed in the signal intensities in the region 455–490 indicates some restriction of motion compared to residues in the C-terminal part of the protein. This restriction of motion could be induced by a transient contact between the MoRE of N_TAIL_ and the surface of the nucleocapsids or by an increased local correlation time of the partially folded MoRE due to a “drag” effect induced by the presence of the nucleocapsid core domain [53].

### Interaction of intact HeV nucleocapsids with XD

**Figure 10:**
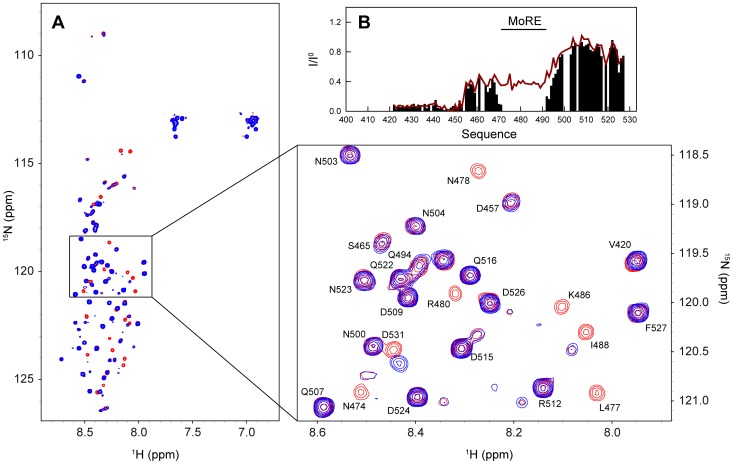
Characterization of the interaction between ^15^N labeled nucleocapsids and unlabeled XD. (A) Superposition of the ^1^H-^15^N HSQC spectra of intact nucleocapsids (red) and intact nucleocapsids with 1.5-fold molar excess of XD (blue). The spectra were obtained at a ^1^H frequency of 600 MHz and 293 K. The expanded region shows the spectral assignment of a number of resonances in the spectrum of the nucleocapsids. The assignment was transferred from the spectrum of the isolated N_TAIL_ domain. (B) Intensity profile (black) of the HSQC spectrum of the nucleocapsids with XD. The profile is calculated as *I*/*I* ^0^, where *I* are the intensities in the spectrum of the nucleocapsids with XD, and *I* ^0^ are the intensities in the spectrum of the isolated N_TAIL_ domain. The red line corresponds to the intensity profile of the nucleocapsid spectrum obtained in the absence of XD (see figure 9).

The observed NMR signals of N_TAIL_ within the nucleocapsids provide a unique probe of nucleocapsid morphology. Our next goal was thus to directly monitor the interaction between the nucleocapsids and XD in order to observe whether any changes in the local conformational behaviour of N_TAIL_ or in the overall morphology of the nucleocapsids occur upon XD binding. The interaction between XD and full-length nucleocapsids of paramyxoviruses had previously not been amenable to structural studies, in particular, MeV nucleocapsids were found to precipitate upon addition of XD *in vitro* (data not shown). In the case of HeV, however, no such behaviour was observed, and we were able to record well-resolved spectra of nucleocapsids complexed with XD (Figure 10A). HSQC spectra of intact nucleocapsids in the absence and presence of a saturating amount of XD superimpose well and show that XD interacts only locally with the MoRE of N_TAIL_ (Figure 10A). The intensity profile of the nucleocapsids in the presence of XD (Figure 10B) closely resembles that of the nucleocapsids alone except that the resonances of the MoRE have disappeared, as is the case in the titration of the isolated N_TAIL_ domain with XD (Figure 6D). Importantly, the intensities outside the MoRE (residues 420–468 and residues 500–527) are not modulated by the presence of XD and no additional peaks are observed (Figure 10B). The fact that we see the same intensities and chemical shifts, and therefore the same conformational signature, for these residues upon interaction with the X domain of the phosphoprotein provides evidence that the environment of N_TAIL_ is effectively identical in the free and XD-bound form of the nucleocapsids. Any major rearrangement or unwinding of the nucleocapsids upon interaction with XD would be expected to change the conformational signature of N_TAIL_. In particular, those N_TAIL_ residues whose motion is evidently strongly restricted within intact nucleocapsids (i.e. residues 401–450) should gain in NMR signal intensity if the nucleocapsids were unwound, which is not observed. The fact that the small residual signals from residues 420–450, interpreted as nucleocapsid edge effects, are preserved indicates additionally that the length distribution of the nucleocapsids remains unchanged. Taken together, our results thus strongly suggest that the helical nucleocapsid remains intact upon interaction with XD.

## Discussion

### Differential flexibility and transient order in HeV N_TAIL_

In this study, we have characterized the X domain of the polymerase-associated phosphoprotein and the N_TAIL_ domain of the nucleoprotein whose interaction is essential for the replication and transcription process. We have provided an atomic resolution structural and dynamic characterization of HeV N_TAIL_ using NMR spectroscopy. From experimental chemical shifts and nuclear relaxation data, we have shown that N_TAIL_ adopts conformations close to a random coil, except for the interaction site with the phosphoprotein that populates up to 50% α-helical conformations in broad agreement with previous global spectroscopic analyses [9]. A similar structural architecture was also observed for N_TAIL_ of two closely related viruses, namely SeV and MeV, whose MoREs adopt a dynamic equilibrium between a completely unfolded state and several specific helical conformations [52], [54]–[57].

To further extend the structural characterization of N_TAIL_ to a physiologically relevant environment, we studied the conformational behaviour and flexibility of N_TAIL_ within intact nucleocapsids. To this end, we expressed and purified intact nucleoprotein that assembles on cellular RNA in *E. coli*, to form helical nucleocapsids. Using solution NMR, N_TAIL_ was found to remain flexible *in situ* supporting a functional role for structural disorder in orchestrating the replicative machinery of *Paramyxoviridae*. The flexibility of N_TAIL_ in the context of the nucleocapsids was found to deviate from that observed for the isolated domain, with a gain of rigidity in the first 50 residues of N_TAIL_. The NMR intensity profile of N_TAIL_ (Figure 9B) bears some resemblance to the profile measured from the homologous N_TAIL_ domain within intact MeV nucleocapsids [52]. In the previous study of MeV, a combination of NMR, electron microscopy and small angle X-ray scattering was used to propose a model whereby MeV N_TAIL_ exfiltrates from the inside to the outside of the helical nucleocapsids via the interstitial space between folded N_CORE_ domains. Indeed, the first 50 amino acids of MeV N_TAIL_ could not be detected by solution NMR due to restricted conformational flexibility as the disordered chain escapes from the inside to the outside of the nucleocapsids. The results obtained here are in agreement with a similar model for the location of N_TAIL_ in HeV nucleocapsids.

Despite the overall similarity in the conformational behaviour of N_TAIL_ within the nucleocapsids of HeV and MeV, the intensity profiles are however strikingly different within their MoREs. In MeV, the MoRE of N_TAIL_ appears to be co-localized on the surface of the nucleocapsids up to 95% of the time, resulting in heavily attenuated signals in the MoRE. We have previously proposed that this co-localization may have functional implications, providing a mechanism by which N_TAIL_ can position the viral polymerase complex close to the viral RNA. In HeV, this co-localization appears weaker, as evidenced by appreciable intensities of the solution NMR signals in the MoRE (Figure 9B). This different location of the MoRE might provide a basis for a different mechanism of recognition by the polymerase complex in the two viruses.

### Insight into the molecular mechanisms underlying the HeV N_TAIL_-XD interaction

Chemical shift titrations suggest that upon interaction with the C-terminal X domain of the phosphoprotein, the MoRE of N_TAIL_ undergoes α-helical folding. This observation is supported by previous studies of this interaction using circular dichroism (CD) and EPR spectroscopy that showed that XD triggers α-helical folding of N_TAIL_ upon binding [7], [8]. On the basis of the changes in the NMR signal intensities of N_TAIL_ upon addition of an increasing amount of XD (Figure 6B, C, D), we suggest that the formation of the N_TAIL_-XD complex relies on a combination of conformational selection and induced folding, where complex formation proceeds via an initial binding of a short helix in the centre of the MoRE that is subsequently extended by α-helical folding of the adjacent residues. The complex between N_TAIL_ and XD appears to be dynamic, as the resonances of the MoRE of N_TAIL_ do not reappear even for large saturating amounts of XD, suggesting helical fraying of the MoRE of N_TAIL_ on the surface of XD.

To obtain further insight into the structural basis of transcription and replication of HeV, we determined the crystal structure of XD. The structure consists of a three-helix bundle arrangement in agreement with previous studies showing that the *Henipavirus* X domains form autonomously folding units adopting an α-helical conformation [7]. NMR chemical shifts and RDCs of XD measured in solution confirmed the triple-helix structure. The structure of HeV XD is very similar to the structure of the XD domains of MeV [58], SeV [59] and mumps virus [60] P proteins with backbone RMSD values of 1.22, 1.34 and 0.88 Å, respectively. Although a comparison of the primary sequences of the X domains of SeV, MeV and HeV shows significant divergence, the hydrophobic residues buried within the XD structure are conserved among the three viruses (Figure S4 in Text S1). The reproduction of an essentially identical hydrophobic core in all three viruses implies that this particular triple-helix arrangement has a strong functional relevance in paramyxoviruses.

Our results show that the two helices α2 and α3 of XD constitute the binding site for the MoRE of N_TAIL_, as also observed in the corresponding N_TAIL_-XD pairs in both MeV and SeV [54], [61]. The interaction between XD and N_TAIL_ in HeV appears to be controlled by a combination of long-range electrostatic forces that correctly orient N_TAIL_ prior to accommodation in the narrow hydrophobic pocket on the surface of XD. The extent to which these long-range electrostatic interactions play a role in complex formation remains however to be established, since previous studies showed that salt concentrations as high as 1 M do not affect the N_TAIL_-XD binding affinity [7]. A comparison of the distribution of hydrophobic and charged residues on the surface of the X domains of SeV, MeV and HeV shows that they all display a hydrophobic pocket of varying size at the interface between helices α2 and α3 (Figure S5 in Text S1). In contrast to the hydrophobic core that is reproduced in all three viruses and provides the stabilizing scaffold for XD, the charge distribution on and around the interaction surface with N_TAIL_ varies significantly over X domains. These differences presumably play a role in the selectivity of the N_TAIL_-XD interactions in SeV, MeV and HeV.

Although high resolution NMR spectroscopy, as well as previous biochemical data [7], suggest that HeV N_TAIL_-XD forms a so-called ‘fuzzy’ complex [62], which is characterized by mobility within the complex, we have nevertheless attempted to model the structure of the complex in terms of a single conformation using chemical shift mapping of the two interacting interfaces. The results (Figure S6 in Text S1) reveal that neither chemical shifts, nor electrostatic interactions, are able to distinguish between the directionality of the N_TAIL_ helix on the surface of XD. The experimental data do not distinguish rotational symmetry about the axis of the N_TAIL_ helix, although two conformations are most probable, both having the hydrophobic face of the MoRE of N_TAIL_ identified in Figure 7 in contact with the hydrophobic interface of XD. In both conformations the two arginines flanking the hydrophobic face on N_TAIL_ interact with acidic patches on the surface of XD. Notably, irrespective of the direction of the MoRE, its optimal position with respect to the observed chemical shift perturbations on the surface of XD is very similar to that observed in the chimeric crystal structure of MeV N_TAIL_-XD (Figure S6 in Text S1).

### Binding of XD to intact nucleocapsids and functional implications for transcription and replication

In paramyxoviruses, genome transcription and replication rely on the N-P interaction that is critical for the recruitment of the polymerase. This interaction is thought to make the viral RNA accessible to the polymerase L through a structural reorganisation of the nucleocapsid. The molecular mechanisms of this critical step remain, however, poorly understood and to the best of our knowledge no such nucleocapsid conformational change induced by a polymerase complex has been observed so far in any *Paramyxoviridae* member. As a step towards a better understanding of the structural and molecular basis of transcription and replication, we have performed the first investigation of XD binding to N_TAIL_ in the context of intact nucleocapsids. Our results show that XD interacts only locally with the MoRE of N_TAIL_ and that no additional residues in N_TAIL_ are involved in the interaction. Importantly, the NMR signal intensities outside the MoRE are not perturbed by the presence of XD (Figure 10B). The similarity of the high-resolution NMR spectroscopic parameters measured in the presence and absence of the partner, which together represent the signature that the N-terminal part of N_TAIL_ remains sterically hindered between turns of the nucleocapsid, indicates that there is no major reorganization of the nucleocapsid upon interaction. Any further opening of the interstitial space between turns of the nucleocapsid helix would be expected to give rise to enhanced flexibility and therefore increased signal intensity, both within the first 50 amino acids and in the 10–15 amino acids immediately following this region. In addition, RNAse digestion experiments did not reveal any hyperchromic effect at 260 nm upon addition of XD (data not shown), indicating that XD does not render the encapsidated RNA more accessible to the solvent.

In combination, these observations provide the first experimental evidence that XD can be accommodated on N_TAIL_ without triggering a nucleocapsid rearrangement that could increase the accessibility of the polymerase complex to the viral genome. We can speculate that the presence of the full-length, tetrameric P protein, the polymerase L and/or additional host factors [63], are necessary to trigger any conformational transition that may occur prior to replication of the viral RNA by the polymerase machinery. Notably, the major inducible heat shock protein 70 (hsp70) was shown to enhance both transcription and replication in MeV and in the closely related canine distemper virus (CDV), with this effect relying on an interaction between hsp70 and N [64]–[70]. Taking into account the fact that hsp70 has been suggested to trigger conformational changes within CDV nucleocapsids [71], [72], it is tempting to speculate that unwinding of the nucleocapsid may require hsp70 also in the case of HeV.

## Materials and Methods

### Expression and purification of N_TAIL_

The HeV N_TAIL_ construct, which encodes residues 401–532 of N, with an N-terminal hexahistidine tag has been described previously [15]. Isotopically labeled (^15^N, ^13^C) HeV N_TAIL_ was prepared by growing transformed *E. coli* T7 pLysS (New England Biolabs) cells at 37°C in minimal M9 medium [73] containing 100 mg/L ampicillin and 34 mg/L chloramphenicol supplemented with ^15^NH_4_Cl (1 g/L) and ^13^C-glucose (2 g/L). A 150 mL preculture grown overnight to saturation in 2YT medium containing 100 mg/L ampicilin and 34 mg/L chloramphenicol (2YT-AC) was harvested, washed in minimal M9 medium, and inoculated into 1 L of minimal M9 medium supplemented with ampicillin and chloramphenicol (M9-AC). The culture was grown at 37°C. When the optical density (OD) at 600 nm reached 0.6, protein expression was induced by the addition of 0.5 mM isopropyl β-D-thiogalactopyranoside (IPTG), and the cells were grown over night at 28°C.

Isotopically ^13^C,^15^N and ^15^N labeled N_TAIL_ samples were purified as previously described [7] except that NMR buffer was used during size exclusion chromatography (SEC), and that an additional purification step was performed: the eluent from SEC was loaded onto an anion exchange MonoQ column (GE, Healthcare) and elution was carried out using a gradient of NaCl (10–1000 mM) in 10 mM Tris/HCl pH 8 buffer. The sample was concentrated using Centricon Plus-20 (molecular cutoff of 5,000 Da) (Millipore) and then dialyzed against NMR buffer. A protease inhibitor cocktail was added to the samples before storage at −20°C. The expression and purification of unlabeled N_TAIL_ has already been described [15]. All purification steps, except for gel filtrations, were carried out at 4°C. The protein concentration was estimated using the previously experimentally determined absorption coefficients [8]. Because N_TAIL_ is devoid of both Trp and Tyr residues, the absorbance was measured at 254 nm using the absorption coefficient experimentally determined at this wavelength [8].

### Expression and purification of nucleocapsids

The HeV N construct, encoding full-length N (residues 1–532), with a hexahistidine tag fused to the C-terminus, has already been described [7]. Isotopically labeled (^15^N) HeV nucleocapsids were prepared by growing transformed *E. coli* T7 pLysS (New England Biolabs) cells according to the protocol described by Marley et al [74]. Briefly, an 80 mL overnight preculture grown to saturation in 2YT-AC was used to inoculate 2 L of the same medium. When the OD at 600 nm was approximately 0.6, the culture was harvested and the pellet was resuspended in 500 mL of M9-AC medium containing ^15^NH_4_Cl (1 g/L) and glucose (2 g/L). After an additional growth step at 37°C for 2 hours, 1 mM IPTG was added and the culture was further incubated at 37°C for 3.5 hours. In all cases, the induced cells were harvested, washed and collected by centrifugation (5,000 g, 10 min). The resulting pellets were frozen at −20°C.

The nucleocapsids were purified by resuspending cellular pellets in 5 volumes (v/w) buffer A (50 mM Tris/HCl pH 8, 300 mM NaCl, 10 mM imidazole, 1 mM phenyl-methyl-sulphonyl-fluoride (PMSF) supplemented with 0.1 mg/mL lysozyme, 10 µg/mL DNAse I, 20 mM MgSO_4_ and protease inhibitor cocktail (Sigma, 1 mL per 25 mL of bacterial lysate). After 30 min incubation with gentle agitation, the cells were disrupted by sonication (using a 750 W sonicator and 4 cycles of 30 s each at 45% power output). The lysate was clarified by centrifugation at 30,000 g for 30 min. The clarified supernatant was loaded onto a 5 mL HisTrap FF column (GE, Healthcare), previously equilibrated in buffer A. Elution was carried out using a gradient of imidazole (10–500 mM) in buffer A. Eluates were analyzed by SDS-PAGE for the presence of N. The fractions of interest were pooled and then injected onto a HiPrep 26/10 desalting column (GE, Healthcare). Elution was carried out in NMR buffer (50 mM BisTris, pH 6, 500 mM NaCl). The sample was concentrated using Centricon Plus-20 (molecular cutoff of 10,000 Da) (Millipore) and protease inhibitor cocktail (1/40) was added. The sample was immediately subjected to NMR and electron microscopy analyses. The concentration of nucleocapsid samples was roughly estimated using the theoretical absorption coefficient at 280 nm as provided by the ProtParam program at the ExPASy server [75].

### Expression and purification of XD

A synthetic gene coding for a slightly enlarged XD domain (residues 654–707) of HeV P was cloned in the pET28a vector and was expressed at 20°C in *E. coli* BL21 Rosetta [DE3] with two N-terminal non-native amino acids (Met and Val) and two C-terminal non-native residues (Leu and Glu) followed by a hexahistidine tag. Selenomethionine-substituted protein was produced by growing the bacterial cells in minimal medium and by adding SeMet before induction (1 mM IPTG) of the heterologous protein expression. The protein fragment was purified by Ni^2+^ affinity chromatography on a His-Select (Sigma) and size exclusion chromatography on a Superdex S75 (GE Healthcare) column in 20 mM Tris buffer at pH 7.5 supplemented with 150 mM NaCl, 50 mM Glu/Arg, 0.5 mM TCEP and an anti-protease cocktail (Roche) (Buffer A).

For producing isotopically (^13^C, ^15^N) labeled XD without a hexahistidine tag for NMR spectroscopic measurements, the gene was subcloned into the pETM40 vector (EMBL) with an N-terminal MBP tag followed by a TEV protease cleavage site. The protein was expressed as a MBP fusion as described [74] and purified by affinity chromatography on an Amylose resin column (NEB) and size exclusion chromatography on a Superdex S200 (GE Healthcare) column in buffer A. After TEV protease cleavage, the XD domain was purified by size exclusion chromatography on a Superdex S75 column in 20 mM Bis-Tris at pH 6, supplemented with 150 mM NaCl and 0.5 mM TCEP. The expression and purification of unlabeled XD were previously described [7].

### NMR spectral assignment of N_TAIL_

The spectral assignment of N_TAIL_ was carried out using a 200 µM ^13^C, ^15^N labeled sample in 20 mM Bis-Tris, pH 6.0 and 500 mM NaCl. A set of six triple resonance BEST-type spectra were acquired at 293 K and a ^1^H frequency of 800 MHz: HNCO, intra-residue HN(CA)CO, HN(CO)CA, intra-residue HNCA, HN(COCA)CB, intra-residue HN(CA)CB [76]. All spectra were acquired with sweep widths of 8.0 (^1^H), 1.9 (^15^N), 1.3 (^13^C′), 2.7 (^13^Cα) and 12.4 kHz (^13^Cβ) where the number of complex points in each dimension was 512 (^1^H), 38 (^15^N), 60 (^13^C′), 105 (^13^Cα) and 122 (^13^Cβ). Spectra were processed using NMRPipe [77] and analyzed using CcpNmr [78] and Sparky [79]. ^1^H-^15^N HSQC resonances were not visible for residues S400, S439, S454 and S463. Secondary chemical shifts and secondary structure propensities were calculated using the random coil chemical shifts from RefDB [80].

### ^15^N relaxation measurements of N_TAIL_

^15^N CPMG *R*_2_ relaxation rates and {^1^H}-^15^N steady state heteronuclear Overhauser effects (nOes) of N_TAIL_ were measured at a ^1^H frequency of 600 MHz and 293 K using standard pulse sequences on a 150 µM ^15^N labeled sample [81]. The ^15^N *R*_2_ relaxation rates were obtained by sampling the decay of the magnetization at the following time points: 0.01, 0.03, 0.05, 0.07, 0.09, 0.13, 0.17, 0.21 and 0.25 s. A repeat of the time point at 0.07 s was measured for the purpose of error estimation. For the heteronuclear nOes, the amide protons were saturated using a 3 s WALTZ16 decoupling scheme that in the reference experiment was replaced by a 3 s delay. The recycle delay in both experiments was set to 2 s. The nOe values were calculated from the ratio between signal intensities in the saturated and the reference experiment, where the standard deviation in the noise was taken as a measure for the error in the signal intensity.

### Titration of ^15^N labeled N_TAIL_ with unlabeled XD

The interaction between N_TAIL_ and XD was studied using a ^15^N labeled sample of N_TAIL_ by addition of an increasing amount of unlabeled XD. The same buffer conditions as described above for N_TAIL_ was used for both proteins. ^1^H-^15^N HSQC spectra were measured at 293 K and a ^1^H frequency of 800 MHz for the following concentrations of [XD]∶[N_TAIL_]: 0.0∶40.0, 3.0∶39.8, 5.9∶39.6, 8.9∶39.4, 11.8∶39.2, 14.6∶39.0, 17.5∶38.8, 23.2∶38.6, 28.8∶38.5, 34.4∶38.3, 45.7∶38.1, 56.9∶37.9, 162.2∶37.2 µM.

### Crystal structure of XD

Crystals of XD were grown at 293 K by the hanging drop vapor diffusion method in 100 mM Bis-Tris buffer, pH 6 containing 50 mM MgCl_2_ and 26–28% PEG 3350. The protein concentration was between 10 and 15 mg/mL. Crystals were optimized by micro-seeding in the same solution. X-ray diffraction data were collected on ESRF beamline ID14-4 [82] using an inverse beam strategy. Data were integrated, scaled and merged with XDS and XSCALE [83]. The anomalously scattering Se atoms were located and initial phases were obtained using SHELX C/D/E [84] as implemented in HKL2MAP [85]. An initial model obtained with ARPwARP [86] from the CCP4 suite [87] was subsequently refined at 1.65 Å resolution with phenix.refine using unmerged Friedel pairs [88] and manually rebuilt after visual inspection in Coot [89]. After multiple cycles of refinement, the final crystallographic R-factor and R_free_ values were 18.8% and 22.4%, respectively. Other statistics are reported in Table S2 in Text S1.

### NMR spectral assignment of XD

The spectral assignment of XD was carried out using a 2.5 mM ^13^C, ^15^N labeled sample in 20 mM Bis-Tris, 50 mM Glu/Arg, 150 mM NaCl at pH 6.0. A set of six triple resonance BEST-type spectra were acquired at 298 K and a ^1^H frequency of 800 MHz: HNCO, intra-residue HN(CA)CO, HN(CO)CA, intra-residue HNCA, HN(COCA)CB, intra-residue HN(CA)CB [76]. All spectra were acquired with sweep widths of 10.0 (^1^H), 1.9 (^15^N), 1.9 (^13^C′), 3.8 (^13^Cα) and 14.0 kHz (^13^Cβ) where the number of complex points in each dimension was 704 (^1^H), 39 (^15^N), 70 (^13^C′), 125 (^13^Cα) and 135 (^13^Cβ). Spectra were processed using NMRPipe [77] and analyzed using CcpNmr [78] and Sparky [79]. The ^1^H-^15^N HSQC spectra of XD with and without 50 mM Glu/Arg were almost identical allowing the transfer of assignments to the spectrum without Glu/Arg buffer.

### Measurement of residual dipolar couplings in XD

Four types of residual dipolar couplings (^1^D_HN-N_, ^1^D_Cα-Hα_, ^1^D_Cα-C′_, ^2^D_HN-C′_) were measured in XD using BEST-type experiments at a ^1^H frequency of 600 MHz and 298 K [90]. The protein was aligned in a suspension of filamentous phages (ASLA Biotech) at 11.5 mg/mL giving rise to a residual deuterium splitting of 7 Hz. All spectra were acquired with sweep widths of 7.5 (^1^H), 1.4 (^15^N), 1.2 (^13^C′) and 2.9 (^13^Cα) where the number of complex points in each dimension was 512 (^1^H), 40 (^15^N), 80 (^13^C′) and 80 (^13^Cα). Spectra were processed using NMRPipe [77] and analyzed using CcpNmr [78]. The alignment tensor was determined by fitting the experimental RDCs to the crystal structure of XD using the program Module [91]. Only the RDCs for residues within regular secondary structure elements were used in the analysis.

### Titration of ^15^N labeled XD with unlabeled N_TAIL_

In order to map the interaction of N_TAIL_ on XD, a titration was carried out using a ^15^N, ^13^C labeled sample of XD by addition of an increasing amount of unlabeled N_TAIL_. The titration was carried out in 20 mM Bis-Tris buffer, 150 mM NaCl at pH 6.0. ^1^H-^15^N HSQC spectra were measured at 298 K and a ^1^H frequency of 800 MHz for the following concentrations of [N_TAIL_]∶[XD]: 0.0∶80.6, 9.5∶80.2, 19.0∶79.9, 28.4∶79.5, 37.7∶79.2, 56.0∶78.5, 74.0∶77.8, 91.7∶77.1, 126.2∶75.8, 159.6∶74.5, 215.3∶72.4, 289.6∶69.5, 358.2∶66.9, 659.3∶55.4 µM. Concentrations were determined by amino acid analysis.

### NMR experiments on recombinant HeV nucleocapsids

A ^1^H-^15^N HSQC spectrum of a 37 µM ^15^N labeled sample of intact nucleocapsids was obtained at 293 K and a ^1^H frequency of 600 MHz. The interaction between the nucleocapsids and XD was studied by the addition of an excess amount of unlabeled XD (55 µM). The ^1^H-^15^N HSQC experiment was repeated for this sample with the same parameters as for the isolated nucleocapsids.

### Negative staining transmission electron microscopy studies

Drops of 2 µL of freshly purified HeV nucleocapsids at 0.05 mg/mL were deposited on evaporated carbon membranes on standard copper grids (300 Meshes). Grids were exposed to plasma glow discharge for 20 seconds, prior to protein deposition in order to increase protein adhesion. Grids were then negatively stained with a 2% uranyl acetate solution and imaged using a TECNAI 120 keV transmission electron microscope. Images were collected on a 3 by 3 cm CCD camera with a 15 µm by 15 µm pixel size.

## Supporting Information

Text S1 This file contains six additional figures showing the assigned HSQC spectra of N_TAIL_ and XD (Figures S1, S2 and S3), comparison of the X domains of Sendai, Measles and Hendra viruses (Figures S4 and S5) and a model of the Hendra virus N_TAIL_-XD complex from NMR chemical shift perturbations (Figure S6). In addition, the file contains four additional tables with chemical shift values of N_TAIL_ and XD (Tables S1 and S3), data collection and refinement statistics of XD (Tables S2) and residual dipolar couplings of XD (Table S4). Crystallographic coordinates and structure factors of XD have been deposited in the Protein Data Bank with accession code 4HEO.
(DOC)
