# Supplementary material for: Atomic Resolution Description of the Interaction between the Nucleoprotein and Phosphoprotein of Hendra Virus
Source: PLoS Pathog. 2013 Sep 26;9(9):e1003631. doi: 10.1371/journal.ppat.1003631 (PMC3784471; doi:10.1371/journal.ppat.1003631)
Supplement: Text S1 — This file contains six additional figures showing the assigned HSQC spectra of NTAIL and XD (Figures S1, S2 and S3), comparison of the X domains of Sendai, Measles and Hendra viruses (Figures S4 and S5) and a model of the Hendra virus NTAIL-XD complex from NMR chemical shift perturbations (Figure S6). In addition, the file contains four additional tables with chemical shift values of NTAIL and XD (Tables S1 and S3), data collection and refinement statistics of XD (Tables S2) and residual dipolar couplings of XD (Table S4). Crystallographic coordinates and structure factors of XD have been deposited in the Protein Data Bank with accession code 4HEO. (DOC) [file ppat.1003631.s001.doc]

**Supporting Information**

**Atomic resolution description of the interaction between the nucleoprotein and phosphoprotein of Hendra virus**

Guillaume Communie^1,2,3,4,5,6†^ , Johnny Habchi^7†^, Filip Yabukarski^4,5,6^, David Blocquel^7^, Robert Schneider^1,2,3^, Nicolas Tarbouriech^4,5,6^, Nicolas Papageorgiou^7^, Rob W.H. Ruigrok^4,5,6^, Marc Jamin^4,5,6^, Malene Ringkjøbing Jensen^1,2,3^*, Sonia Longhi^7^* and Martin Blackledge^1,2,3^

^1^Université Grenoble Alpes, Institut de Biologie Structurale (IBS), F-38027 Grenoble, France

^2^CEA, DSV, IBS, F-38027 Grenoble, France

^3^CNRS, IBS, F-38027 Grenoble, France

^4^Université Grenoble Alpes, UVHCI, Grenoble, France

^5^CNRS, UVHCI, Grenoble, France

^6^Unit for Virus Host Cell Interactions, Université Grenoble Alpes-EMBL-CNRS, Grenoble, France

^7^CNRS and Aix-Marseille Université, Architecture et Fonction des Macromolécules Biologiques, UMR 7257, 13288 Marseille, France

† These authors contributed equally to this work

* To whom correspondence should be addressed

Malene Ringkjøbing Jensen

E-mail: malene.ringkjobing-jensen@ibs.fr

Tel: (33) 4 38 78 47 80**,** Fax: (33) 4 38 78 54 94

Sonia Longhi

E-mail: Sonia.Longhi@afmb.univ-mrs.fr

Tel: (33) 4 91 82 55 80**,** Fax: (33) 4 91 26 67 20

**Figure S1:** **Assignment of the ^1^H-^15^N HSQC spectrum of HeV N_TAIL_.** The spectrum was acquired at 293 K in 20 mM Bis-Tris and 500 mM NaCl at pH 6.0.

**
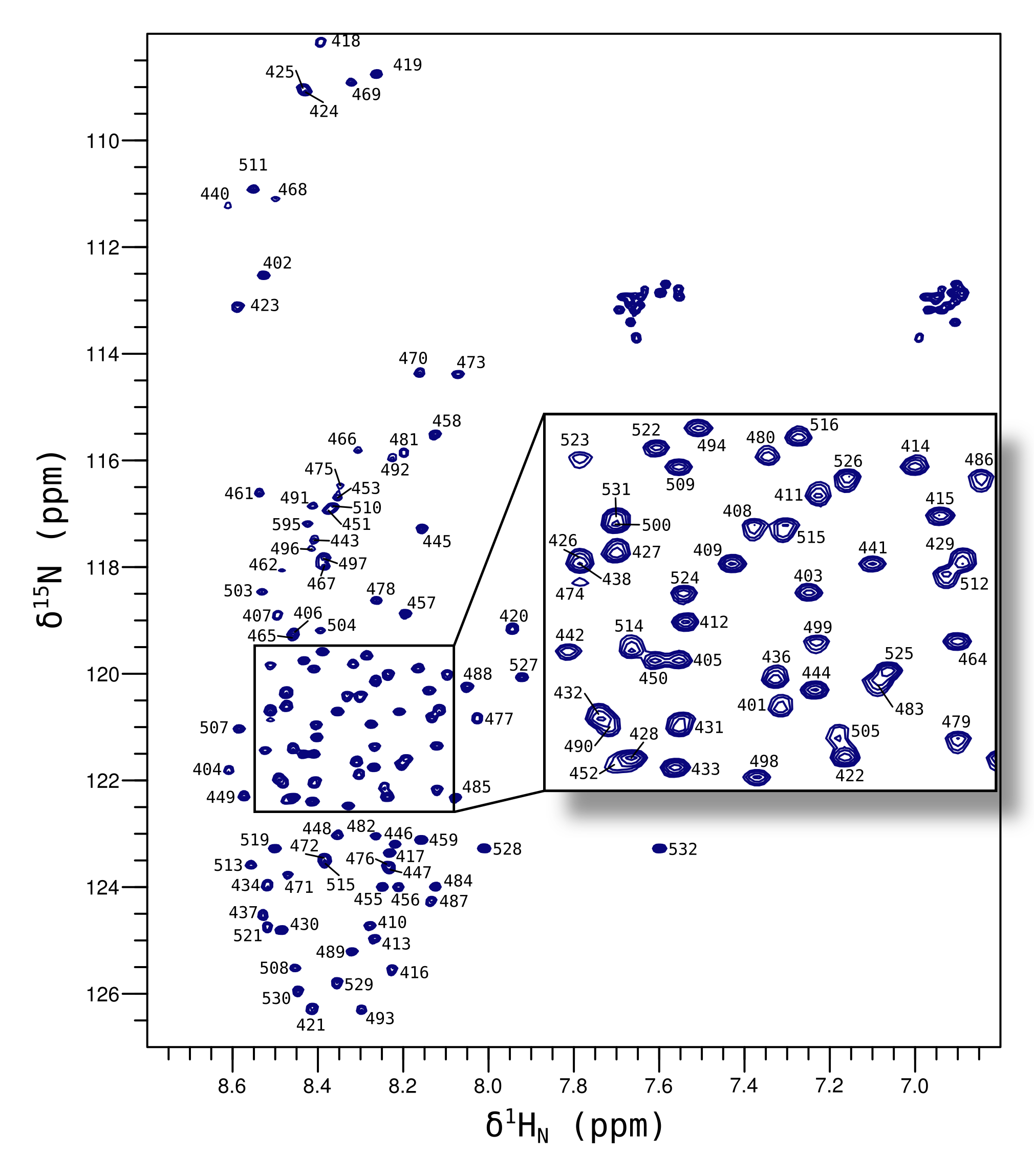
**

**Figure S2:** **Assignment of the ^1^H-^15^N HSQC spectrum of HeV XD.** The spectrum was acquired at 298 K in 20 mM Bis-Tris, 50 mM Arg/Glu, 150 mM NaCl at pH 6.0.

**
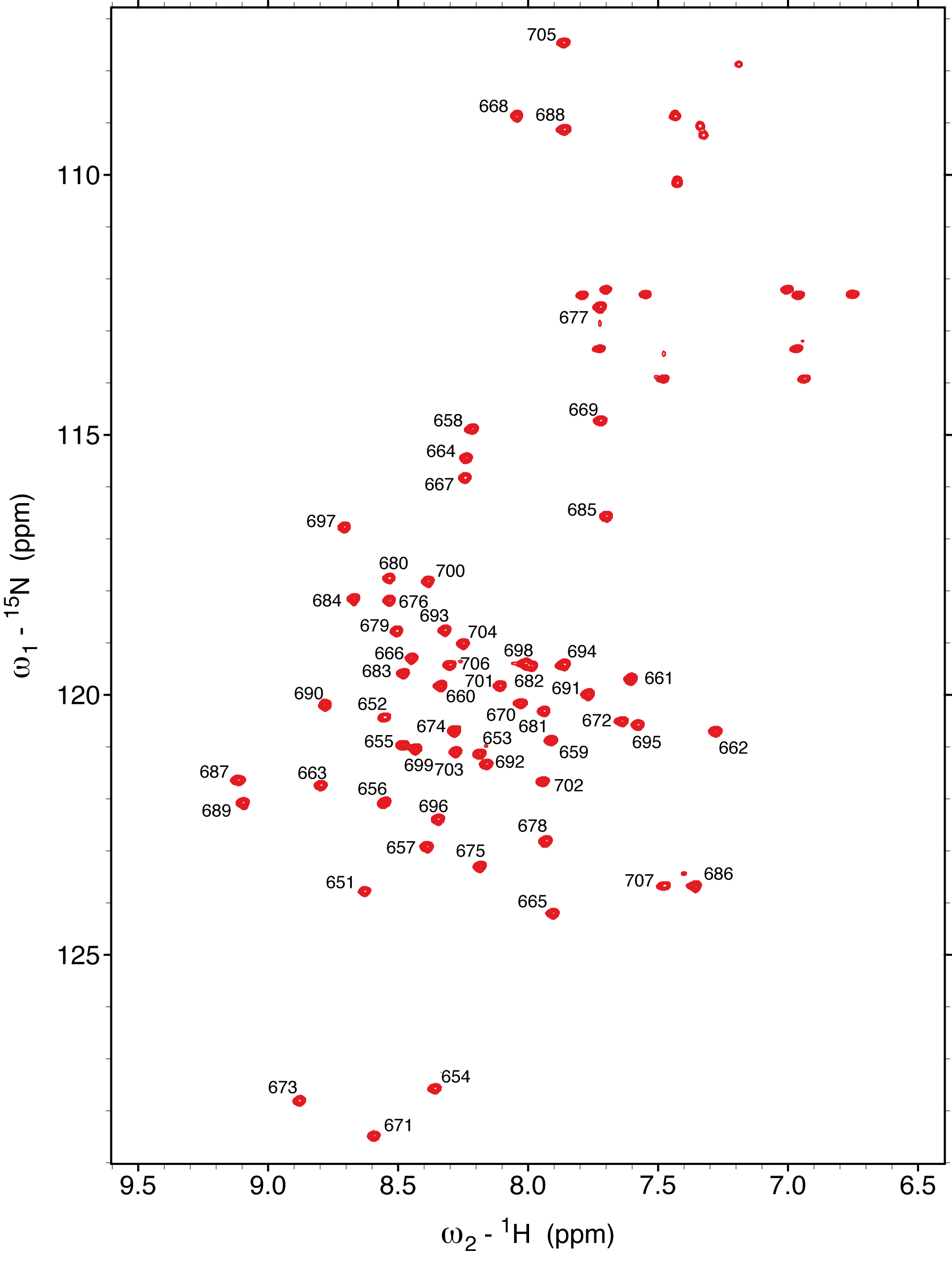
**

**Figure S3:** **Expanded region of the ^1^H-^15^N HSQC spectrum of HeV N_TAIL_.** The spectrum was obtained in the absence of XD (red) and in the presence of 30% (molar fraction) of XD (blue). The observed chemical shift changes correspond to those reported in Figure 5B.

**
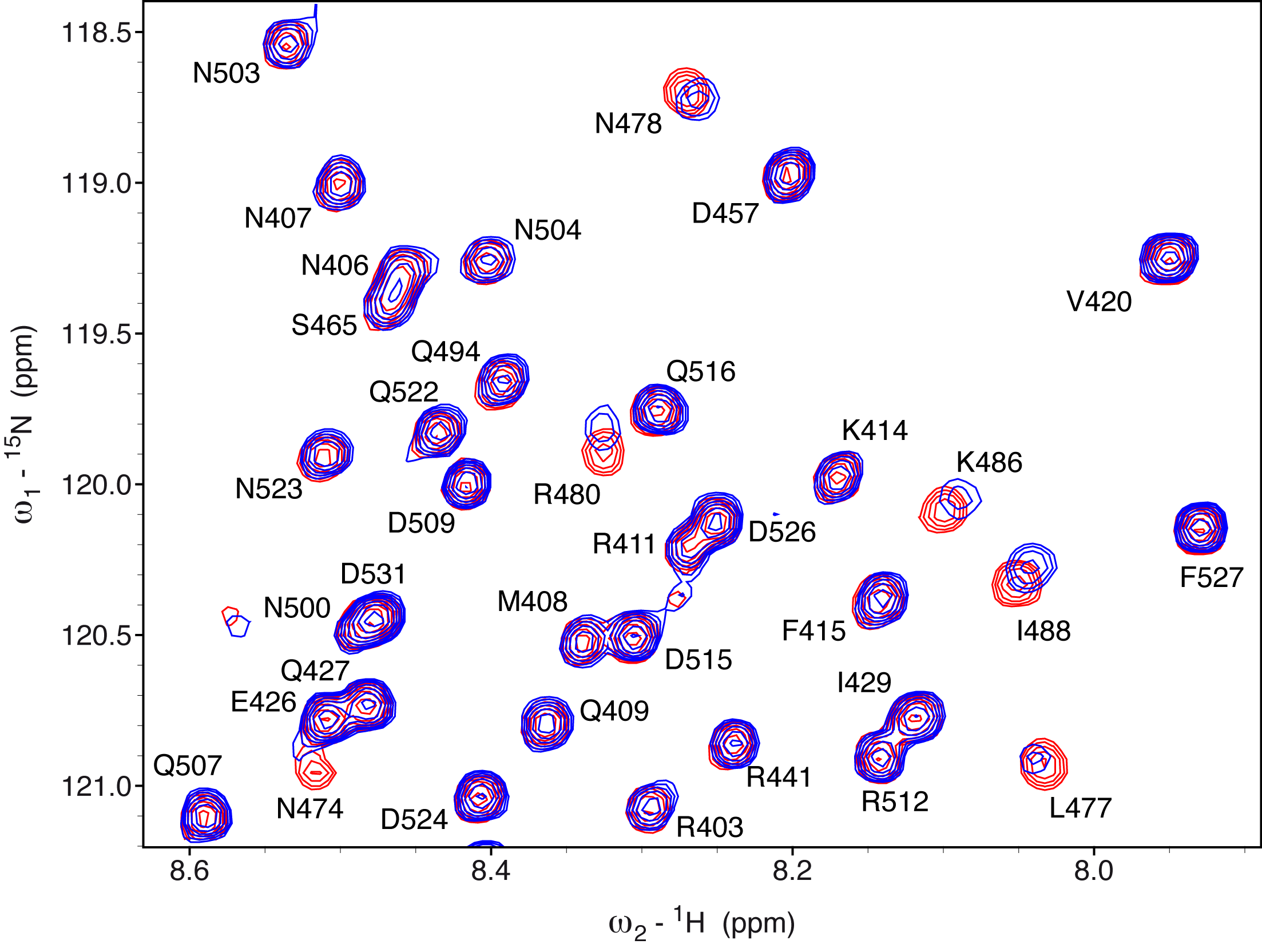
**

**Figure S4:** **The three-helix arrangement in the C-terminal domains of three paramyxoviruses.** (A) Sequence comparison of Sendai, Measles and Hendra virus X domains with conserved hydrophobic, positively charged, negatively charged and other residues indicated in beige, blue, red and gray colour respectively. (B) Mapping of conserved residues on the crystal structure of Hendra virus XD using the colour coding from (A). Left: front view of XD in cartoon representation. Middle: front view of XD in surface representation. Right: top view of XD in cartoon representation. The majority of conserved residues reside in the hydrophobic core of XD, while almost no conserved residues are located on the surface of XD.


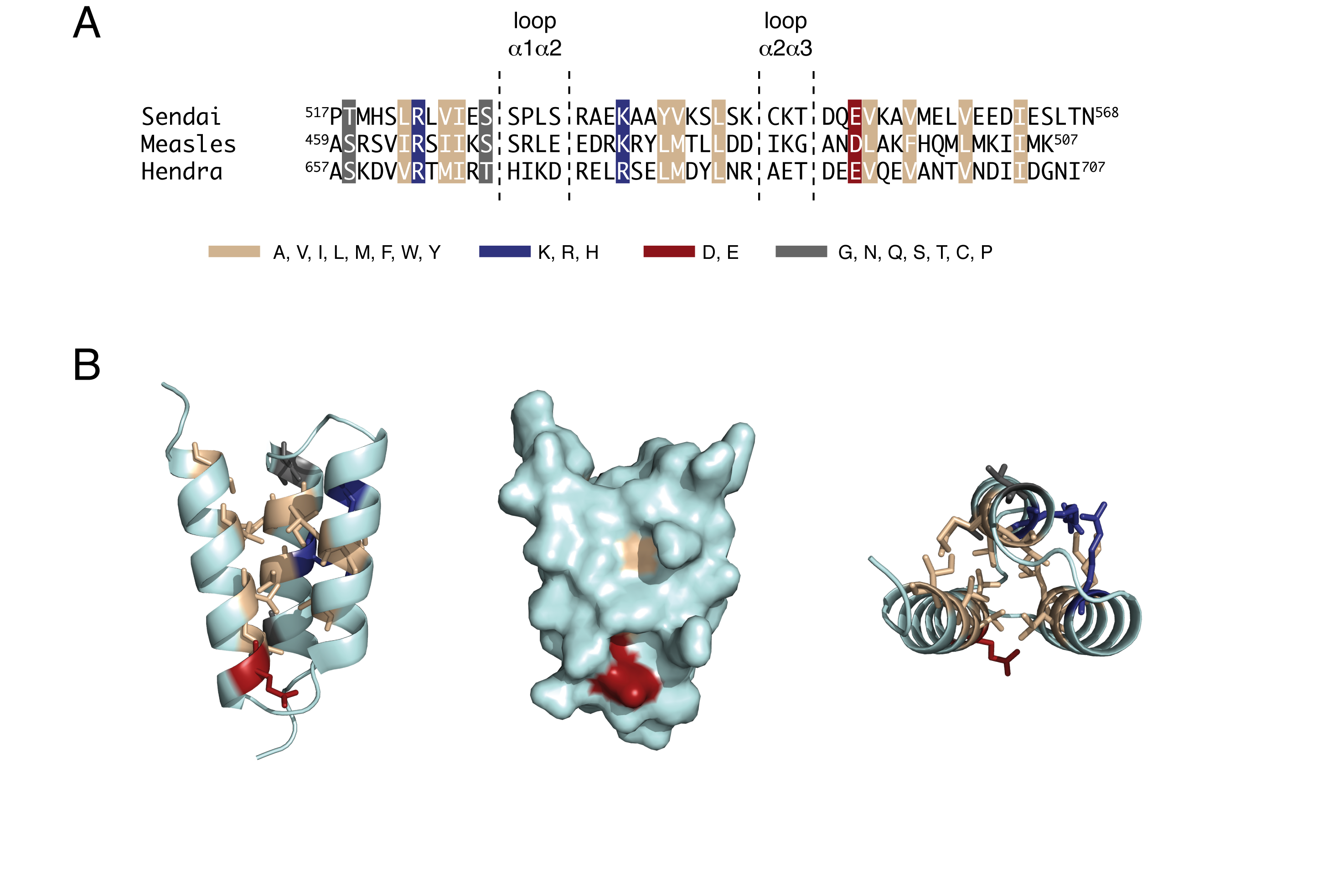


**Figure S5:** **Comparison of the N_TAIL_-XD interaction surfaces of Sendai, Measles and Hendra viruses.** (A) Surface representation of the X domains displaying the location of hydrophobic (beige), negatively charged (red) and positively charged (blue) residues. The orientation of the X domains is the same as shown in Figures 3 and 7. (B) Helical wheel representations of the MoRE of the N_TAIL_ domains with the same color-coding as in (A). The orientation of the wheel of the MoRE of HeV N_TAIL_ is the same as shown in Figure 7. For the sake of clarity, the wheels of SeV and MeV are shown in the same orientation as that of HeV.


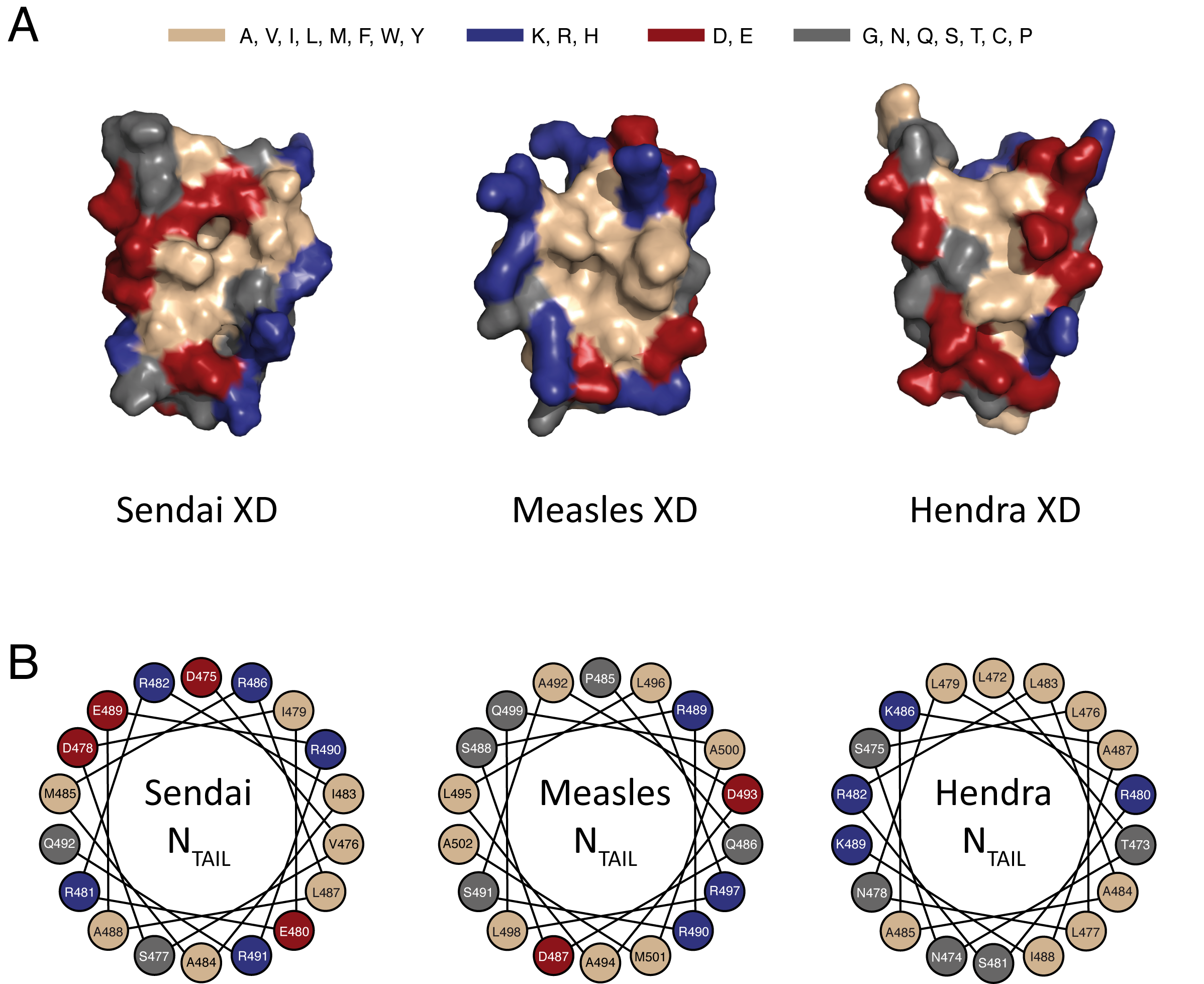


**Figure S6:** **Docking of N_TAIL_ on the surface of XD using experimental chemical shift perturbations as restraints.** (A) Observed chemical shift perturbations in XD used as ambiguous restraints in the docking calculations (purple). All residues between 471 and 489 were used as restraints in N_TAIL_. All restraints were combined as ambiguous distance restraints using standard procedures and used in a torsion-angle molecular dynamics refinement (initial sampling and annealing at 4000 K, followed by slow cooling to 100 K, and refined using Cartesian dynamics and minimization – all calculations were performed using the program CNS-Sculptor). 100 initial structures were created with N_TAIL_ placed randomly on the surface of a sphere of radius 40 Å with the centre of mass of XD at the centre. Typical solutions are shown in (B) and (C), with the hydrophobic surfaces shown in Figure 7 in direct contact, and the basic (N_TAIL_) and acidic (XD) residues stabilizing the interaction. All constraints and biophysical considerations are in agreement with two directions of N_TAIL_ on the surface of XD (up-down and down-up). (D) Comparison of the best-fitting solution with the X-ray crystallographic coordinates of MeV N_TAIL_-XD (orange and blue). Note that in this particular conformation the orientation of HeV N_TAIL_ is inverted with respect to the orientation in MeV.


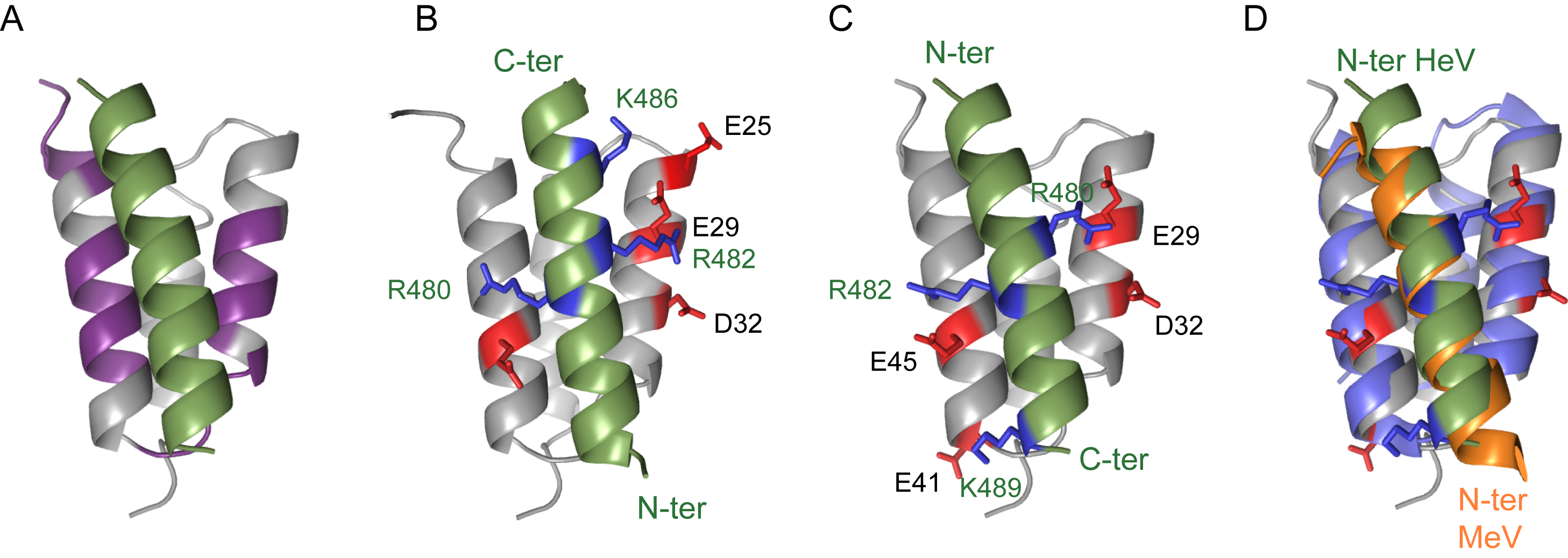


**Table S1:** **Chemical shifts (ppm) of Hendra virus N_TAIL_.** The chemical shifts were obtained at 293 K in 50 mM Bis-Tris, 500 mM NaCl at pH 6.0.

HN N C’ Cα Cβ

------------------------------------------------------------

400 Ser - - 174.47 58.32 63.88

401 Val 8.30 121.85 176.68 62.54 32.72

402 Gly 8.53 112.59 174.05 45.23 -

403 Arg 8.29 121.03 176.55 56.29 30.92

404 Gln 8.61 121.82 175.67 55.85 29.44

405 Asp 8.42 121.54 176.10 54.37 41.24

406 Asn 8.46 119.28 175.31 53.55 38.78

407 Asn 8.50 118.94 175.67 53.71 38.68

408 Met 8.33 120.49 176.72 56.28 32.39

409 Gln 8.36 120.74 176.27 56.55 29.11

410 Ala 8.28 124.75 178.21 53.03 19.05

411 Arg 8.27 120.15 176.73 56.82 30.79

412 Glu 8.41 121.21 176.66 56.97 30.15

413 Ala 8.27 124.99 178.00 53.01 19.14

414 Lys 8.17 119.94 176.59 56.75 32.91

415 Phe 8.14 120.32 175.76 57.78 39.66

416 Ala 8.23 125.59 177.33 52.42 19.41

417 Ala 8.23 123.39 178.40 52.87 19.11

418 Gly 8.40 108.19 174.80 45.47 -

419 Gly 8.27 108.80 173.99 45.28 -

420 Val 7.95 119.23 176.01 62.28 32.85

421 Leu 8.42 126.30 177.24 55.06 42.28

422 Val 8.24 122.30 176.70 62.50 32.76

423 Gly 8.59 113.15 174.72 45.37 -

424 Gly 8.43 109.12 174.82 45.35 -

425 Gly 8.44 109.11 174.40 45.25 -

426 Glu 8.51 120.73 176.64 56.86 30.14

427 Gln 8.48 120.64 175.58 55.74 29.80

428 Asp 8.47 122.37 175.93 54.43 41.10

429 Ile 8.12 120.74 175.83 61.02 39.26

430 Asp 8.49 124.81 176.18 54.33 41.34

431 Glu 8.41 122.05 176.42 56.63 30.56

432 Glu 8.49 121.96 176.33 56.49 30.40

433 Glu 8.41 122.39 176.24 56.38 30.66

434 Glu 8.52 123.95 174.51 54.38 29.82

435 Pro - - 177.02 63.09 32.02

436 Ile 8.31 121.66 176.37 61.37 38.92

437 Glu 8.52 124.59 176.30 56.60 30.15

438 His 8.51 120.74 - - -

439 Ser - - 175.23 58.85 63.85

440 Gly 8.61 111.28 174.37 45.47 -

441 Arg 8.23 120.83 176.53 56.38 30.77

442 Gln 8.53 121.51 176.09 55.96 29.47

443 Ser 8.41 117.50 174.73 58.46 63.84

444 Val 8.28 121.78 176.37 62.65 32.70

445 Thr 8.16 117.33 174.20 62.10 69.82

446 Phe 8.23 123.30 175.44 58.00 39.76

447 Lys 8.24 123.63 176.23 56.38 33.13

448 Arg 8.36 123.05 176.46 56.54 30.74

449 Glu 8.57 122.33 176.76 56.87 30.02

450 Met 8.43 121.55 176.47 55.69 32.82

451 Ser 8.38 116.94 174.84 58.62 63.79

452 Met 8.47 122.39 176.65 55.85 32.73

453 Ser 8.36 116.73 174.80 58.84 63.89

454 Ser - - 174.80 58.58 63.84

455 Leu 8.25 124.01 177.63 55.57 42.19

456 Ala 8.22 124.02 177.68 52.95 19.23

457 Asp 8.20 118.92 176.27 54.52 41.18

458 Ser 8.13 115.57 174.17 58.39 64.04

459 Val 8.16 123.15 174.60 60.04 32.55

460 Pro - - 177.21 63.38 32.19

461 Ser 8.54 116.62 174.93 58.64 63.84

462 Ser 8.49 118.10 - 58.78 63.85

463 Ser - - 174.54 58.53 63.86

464 Val 8.12 121.37 176.38 62.42 32.77

465 Ser 8.46 119.32 175.18 58.39 64.00

466 Thr 8.31 115.85 174.77 61.90 69.93

467 Ser 8.39 117.94 175.19 58.61 63.89

468 Gly 8.50 111.17 174.80 45.57 -

469 Gly 8.33 108.97 174.47 45.36 -

470 Thr 8.17 114.41 174.67 62.12 70.01

471 Arg 8.48 123.85 176.48 56.50 30.59

472 Leu 8.39 123.52 177.75 55.47 42.20

473 Thr 8.07 114.42 174.65 62.24 69.81

474 Asn 8.51 120.82 175.82 53.90 38.61

475 Ser 8.35 116.53 175.29 59.42 63.62

476 Leu 8.24 123.59 178.10 56.21 41.92

477 Leu 8.03 120.87 177.88 56.20 42.13

478 Asn 8.27 118.66 175.86 53.90 38.57

479 Leu 8.12 122.24 178.14 56.70 42.16

480 Arg 8.32 119.85 177.68 57.66 30.24

481 Ser 8.21 115.92 175.55 59.59 63.41

482 Arg 8.26 123.14 177.45 57.47 30.47

483 Leu 8.20 121.71 178.12 56.03 42.05

484 Ala 8.13 123.98 178.21 53.13 18.93

485 Ala 8.08 122.37 178.46 53.18 18.93

486 Lys 8.10 120.06 176.66 56.90 32.99

487 Ala 8.14 124.32 178.27 52.95 19.04

488 Ile 8.05 120.29 176.87 61.68 38.65

489 Lys 8.32 125.25 176.90 56.83 32.93

490 Glu 8.49 122.01 176.87 56.95 30.25

491 Ser 8.41 116.88 175.17 58.74 63.79

492 Thr 8.23 116.00 174.68 62.12 69.85

493 Ala 8.30 126.34 177.98 52.96 19.18

494 Gln 8.39 119.61 176.36 56.07 29.45

495 Ser 8.43 117.26 174.92 58.56 63.95

496 Ser 8.41 117.60 174.95 58.72 63.83

497 Ser 8.39 117.89 174.70 58.74 63.83

498 Glu 8.33 122.55 176.41 56.78 30.18

499 Arg 8.27 121.45 175.86 56.17 30.85

500 Asn 8.48 120.40 172.37 51.52 38.85

502 Pro - - 176.78 63.20 32.04

503 Asn 8.53 118.46 174.90 53.23 38.80

504 Asn 8.40 119.23 174.82 53.14 38.89

505 Arg 8.24 122.23 174.17 54.24 30.07

506 Pro - - 177.04 63.29 32.10

507 Gln 8.59 121.08 176.03 55.89 29.53

508 Ala 8.45 125.58 177.54 52.76 19.36

509 Asp 8.41 119.95 176.59 54.32 41.31

510 Ser 8.37 116.92 175.31 58.88 63.87

511 Gly 8.55 110.96 174.08 45.42 -

512 Arg 8.13 120.80 176.52 56.13 30.95

513 Lys 8.55 123.66 176.49 56.38 32.99

514 Asp 8.46 121.45 176.07 54.51 41.16

515 Asp 8.31 120.48 176.27 54.48 41.02

516 Gln 8.29 119.71 175.91 55.59 29.62

517 Glu 8.39 123.59 174.47 54.51 29.62

518 Pro - - 176.68 63.04 32.16

519 Lys 8.50 123.27 174.64 54.05 32.43

520 Pro - - 176.69 63.08 32.15

521 Ala 8.52 124.79 177.85 52.49 19.29

522 Gln 8.43 119.81 175.71 55.95 29.52

523 Asn 8.51 119.86 174.98 53.28 39.13

524 Asp 8.41 120.99 176.55 54.79 41.02

525 Leu 8.20 121.67 177.40 55.62 42.16

526 Asp 8.24 120.05 175.90 54.60 41.00

527 Phe 7.93 120.08 175.36 57.87 39.68

528 Val 8.01 123.29 175.71 62.36 32.78

529 Arg 8.36 125.82 175.78 56.04 30.94

530 Ala 8.44 125.98 177.24 52.36 19.64

531 Asp 8.48 120.40 175.23 54.37 40.90

532 Val 7.60 123.31 174.65 63.55 33.39

------------------------------------------------------------

**Table S2: Data collection and refinement statistics of HeV XD.**

|  | **Crystal** |
| --- | --- |
| **Data collection** |  |
| Wavelength (Å) | 0.9393 |
| Space group | P12_1_1 |
| Unit cell  a, b, c (Å)  α, β, γ (°) | 21.49, 43.28, 51.26  90.00, 79.78, 90.00 |
| Resolution^a^ (Å) | 32.85 - 1.65 (1.80 - 1.65) |
| I/σ(I) ^a^ | 14.24 (1.89) |
| Rsym^a^ | 0.037 (0.546) |
| Completeness^a^ (%) | 98.6 (98.7) |
| Multiplicity^a^ | 2.7 (2.48) |
| Total reflections^b^ | 58428 |
| **Refinement** |  |
| Resolution^a^ (Å) | 32.85 - 1.65 (1.72 - 1.65) |
| R-factor^a^ | 0.188 (0.306) |
| R-free^a^ | 0.224 (0.306) |
| Number of atoms   macromolecules  ligands   water | 1010  973 2 35 |
| Number of protein residues | 116 |
| R.m.s. deviations  bond lengths (Å)  bond angles (°) | 0.012 1.12 |
| Ramachandran favored (%) | 99 |
| Ramachandran allowed (%) | 1 |
| Average B-factor   macromolecules   solvent | 37.20 36.90 44.80 |

^a^Values in parentheses are for highest-resolution shell.

^b^Friedel pairs unmerged

**Table S3:** **Chemical shifts (ppm) of Hendra virus XD.** The chemical shifts were obtained at 298 K in 20 mM Bis-Tris, 50 mM Arg/Glu, 150 mM NaCl at pH 6.0.

HN N C’ Cα Cβ

-----------------------------------------------

XXX Gly - - 178.69 43.33 -

XXX Ala 8.62 123.77 177.47 52.50 19.56

XXX Met 8.55 120.43 175.84 55.25 33.27

XXX Val 8.18 121.13 175.81 62.15 33.03

-----------------------------------------------

654 Ala 8.35 127.57 177.05 52.23 19.27

655 Asp 8.48 120.97 176.44 53.39 41.73

656 Asp 8.55 122.07 177.72 56.77 41.12

657 Ala 8.38 122.92 180.26 54.85 18.18

658 Ser 8.21 114.88 177.08 60.98 62.87

659 Lys 7.91 120.87 178.36 60.53 32.84

660 Asp 8.33 119.82 178.71 57.96 40.65

661 Val 7.60 119.69 178.78 66.44 32.00

662 Val 7.27 120.70 177.80 65.86 30.97

663 Arg 8.79 121.74 178.65 61.05 30.14

664 Thr 8.23 115.44 176.87 66.85 68.59

665 Met 7.90 124.20 178.47 59.64 33.38

666 Ile 8.44 119.29 177.19 65.84 38.71

667 Arg 8.24 115.82 178.47 59.75 30.62

668 Thr 8.04 108.86 175.73 64.63 70.33

669 His 7.72 114.73 174.75 57.50 31.80

670 Ile 8.02 120.16 175.43 58.98 34.54

671 Lys 8.59 128.48 176.67 57.43 32.76

672 Asp 7.63 120.51 176.22 54.34 42.68

673 Arg 8.87 127.80 178.49 59.93 30.31

674 Glu 8.28 120.69 179.07 59.53 28.82

675 Leu 8.18 123.29 178.59 57.29 41.63

676 Arg 8.53 118.18 177.78 60.92 30.83

677 Ser 7.72 112.54 176.69 62.22 62.88

678 Glu 7.92 122.80 178.92 59.35 29.61

679 Leu 8.50 118.77 179.12 57.89 42.26

680 Met 8.53 117.75 178.59 57.84 31.44

681 Asp 7.93 120.31 178.58 57.70 40.76

682 Tyr 7.98 119.44 179.05 59.41 37.76

683 Leu 8.47 119.58 178.82 57.49 41.98

684 Asn 8.66 118.15 177.24 55.81 37.85

685 Arg 7.69 116.56 176.27 56.96 31.01

686 Ala 7.35 123.68 177.16 53.07 19.19

687 Glu 9.11 121.64 177.11 57.02 32.49

688 Thr 7.85 109.12 175.08 59.42 72.38

689 Asp 9.09 122.08 178.75 57.67 40.25

690 Glu 8.77 120.20 179.27 60.08 29.23

691 Glu 7.76 119.99 179.75 59.17 30.57

692 Val 8.15 121.33 177.63 67.61 31.55

693 Gln 8.31 118.75 178.01 58.00 27.90

694 Glu 7.85 119.41 179.72 59.56 29.10

695 Val 7.57 120.57 177.90 66.66 30.94

696 Ala 8.34 122.39 179.59 55.79 17.83

697 Asn 8.70 116.77 177.54 55.88 37.66

698 Thr 8.00 119.39 176.15 67.16 68.74

699 Val 8.43 121.04 177.36 67.28 31.42

700 Asn 8.38 117.81 177.16 56.59 38.29

701 Asp 8.10 119.82 178.82 57.50 40.20

702 Ile 7.94 121.66 179.46 64.97 38.38

703 Ile 8.27 121.10 178.16 64.95 38.22

704 Asp 8.24 119.01 177.04 55.48 40.79

705 Gly 7.86 107.45 174.60 45.86 -

706 Asn 8.29 119.42 174.19 53.35 38.97

707 Ile 7.47 123.67 171.81 62.76 39.91

-----------------------------------------------

**Table S4:** **Residual dipolar couplings (Hz) of Hendra virus XD.** The couplings were obtained in filamentous phages (11.5 mg/mL) at 298 K in 20 mM Bis-Tris, 50 mM Arg/Glu, 150 mM NaCl at pH 6.0.

RDC Error

-------------------------------

654 N 654 HN 2.16 1.00

655 N 655 HN 1.32 1.00

656 N 656 HN 9.71 1.00

657 N 657 HN 6.43 1.00

658 N 658 HN 7.13 1.00

659 N 659 HN 13.14 1.00

660 N 660 HN 12.30 1.00

661 N 661 HN -1.33 1.00

662 N 662 HN 9.99 1.00

663 N 663 HN 12.65 1.00

664 N 664 HN 5.03 1.00

665 N 665 HN 1.18 1.00

666 N 666 HN 12.09 1.00

667 N 667 HN 11.81 1.00

668 N 668 HN -3.71 1.00

669 N 669 HN 5.24 1.00

670 N 670 HN 11.88 1.00

671 N 671 HN -2.31 1.00

672 N 672 HN -13.99 1.00

673 N 673 HN -1.75 1.00

674 N 674 HN 13.21 1.00

675 N 675 HN 5.73 1.00

676 N 676 HN -1.54 1.00

677 N 677 HN 8.04 1.00

678 N 678 HN 12.02 1.00

679 N 679 HN 0.97 1.00

680 N 680 HN -0.21 1.00

681 N 681 HN 11.25 1.00

682 N 682 HN 9.64 1.00

683 N 683 HN -0.77 1.00

684 N 684 HN 6.01 1.00

685 N 685 HN 14.47 1.00

686 N 686 HN -6.58 1.00

687 N 687 HN -7.27 1.00

688 N 688 HN 10.76 1.00

689 N 689 HN 6.99 1.00

690 N 690 HN 6.29 1.00

691 N 691 HN 14.96 1.00

692 N 692 HN 8.80 1.00

693 N 693 HN 4.33 1.00

694 N 694 HN 12.44 1.00

695 N 695 HN 14.68 1.00

696 N 696 HN 9.64 1.00

697 N 697 HN 6.11 1.00

698 N 698 HN 14.61 1.00

699 N 699 HN 14.19 1.00

700 N 700 HN 7.13 1.00

701 N 701 HN 10.83 1.00

702 N 702 HN 15.94 1.00

703 N 703 HN 10.13 1.00

704 N 704 HN 5.59 1.00

705 N 705 HN 7.48 1.00

706 N 706 HN 6.92 1.00

707 N 707 HN -3.78 1.00

-------------------------------

654 CA 654 HA -1.53 2.40

655 CA 655 HA -17.14 2.40

656 CA 656 HA -10.97 2.40

657 CA 657 HA 32.27 2.40

658 CA 658 HA -15.08 2.40

659 CA 659 HA -0.99 2.40

660 CA 660 HA 2.06 2.40

661 CA 661 HA 26.88 2.40

662 CA 662 HA -24.39 2.40

663 CA 663 HA -19.37 2.40

664 CA 664 HA 37.68 2.40

665 CA 665 HA -10.06 2.40

666 CA 666 HA -10.87 2.40

667 CA 667 HA -15.06 2.40

668 CA 668 HA 45.81 2.40

669 CA 669 HA -5.07 2.40

670 CA 670 HA -12.63 2.40

671 CA 671 HA 2.65 2.40

672 CA 672 HA 18.28 2.40

673 CA 673 HA -21.31 2.40

674 CA 674 HA -19.59 2.40

675 CA 675 HA 16.84 2.40

676 CA 676 HA 12.81 2.40

677 CA 677 HA -17.29 2.40

678 CA 678 HA -22.92 2.40

679 CA 679 HA 44.36 2.40

680 CA 680 HA -11.49 2.40

681 CA 681 HA -14.23 2.40

682 CA 682 HA -2.99 2.40

683 CA 683 HA 37.34 2.40

684 CA 684 HA -18.20 2.40

685 CA 685 HA -23.15 2.40

686 CA 686 HA 27.01 2.40

687 CA 687 HA 32.32 2.40

688 CA 688 HA -26.48 2.40

689 CA 689 HA 43.30 2.40

690 CA 690 HA -20.24 2.40

691 CA 691 HA -10.52 2.40

692 CA 692 HA 13.41 2.40

693 CA 693 HA 15.48 2.40

694 CA 694 HA -3.47 2.40

695 CA 695 HA -23.10 2.40

696 CA 696 HA 42.49 2.40

697 CA 697 HA -17.00 2.40

698 CA 698 HA 5.19 2.40

699 CA 699 HA -4.68 2.40

700 CA 700 HA 28.16 2.40

701 CA 701 HA -9.62 2.40

702 CA 702 HA -18.16 2.40

703 CA 703 HA 24.72 2.40

704 CA 704 HA 1.88 2.40

706 CA 706 HA -13.49 2.40

-------------------------------

654 C 654 CA 1.20 0.25

655 C 655 CA 1.10 0.25

656 C 656 CA -1.60 0.25

657 C 657 CA -1.20 0.25

658 C 658 CA -0.80 0.25

659 C 659 CA 2.80 0.25

660 C 660 CA -2.90 0.25

661 C 661 CA -1.90 0.25

662 C 662 CA 2.50 0.25

663 C 663 CA 0.30 0.25

664 C 664 CA -2.50 0.25

665 C 665 CA -1.70 0.25

666 C 666 CA 3.90 0.25

667 C 667 CA -1.90 0.25

668 C 668 CA -3.20 0.25

669 C 669 CA -1.80 0.25

670 C 670 CA 3.50 0.25

671 C 671 CA -1.50 0.25

672 C 672 CA -2.40 0.25

673 C 673 CA 1.10 0.25

674 C 674 CA 3.00 0.25

675 C 675 CA -2.80 0.25

676 C 676 CA -2.30 0.25

677 C 677 CA 3.90 0.25

678 C 678 CA -1.10 0.25

679 C 679 CA -2.40 0.25

680 C 680 CA -1.20 0.25

681 C 681 CA 4.30 0.25

682 C 682 CA -2.10 0.25

683 C 683 CA -2.90 0.25

684 C 684 CA 1.60 0.25

685 C 685 CA 2.40 0.25

686 C 686 CA -3.20 0.25

687 C 687 CA 0.50 0.25

688 C 688 CA -2.30 0.25

689 C 689 CA -1.70 0.25

690 C 690 CA 0.50 0.25

691 C 691 CA 2.60 0.25

692 C 692 CA -1.70 0.25

693 C 693 CA -2.30 0.25

694 C 694 CA 2.70 0.25

695 C 695 CA -1.50 0.25

696 C 696 CA -1.40 0.25

697 C 697 CA -1.60 0.25

698 C 698 CA 2.70 0.25

699 C 699 CA -2.00 0.25

700 C 700 CA -1.90 0.25

701 C 701 CA 1.40 0.25

702 C 702 CA 0.30 0.25

703 C 703 CA -0.80 0.25

704 C 704 CA -2.00 0.25

705 C 705 CA 2.60 0.25

706 C 706 CA -0.90 0.25

-------------------------------

654 C 655 HN -1.60 0.40

655 C 656 HN -0.30 0.40

656 C 657 HN -2.10 0.40

657 C 658 HN 3.60 0.40

658 C 659 HN -3.30 0.40

659 C 660 HN -3.80 0.40

660 C 661 HN 4.90 0.40

661 C 662 HN -0.10 0.40

662 C 663 HN -4.80 0.40

663 C 664 HN -3.60 0.40

664 C 665 HN 6.80 0.40

665 C 666 HN -4.60 0.40

666 C 667 HN -3.90 0.40

667 C 668 HN 2.20 0.40

668 C 669 HN 4.80 0.40

669 C 670 HN -4.50 0.40

670 C 671 HN -5.00 0.40

671 C 672 HN 6.60 0.40

672 C 673 HN 5.10 0.40

673 C 674 HN -4.20 0.40

674 C 675 HN -4.80 0.40

675 C 676 HN 5.90 0.40

676 C 677 HN -0.60 0.40

677 C 678 HN -4.50 0.40

678 C 679 HN -2.50 0.40

679 C 680 HN 6.80 0.40

680 C 681 HN -3.80 0.40

681 C 682 HN -4.10 0.40

682 C 683 HN 3.00 0.40

683 C 684 HN 2.30 0.40

684 C 685 HN -3.00 0.40

685 C 686 HN -2.30 0.40

686 C 687 HN 6.60 0.40

687 C 688 HN -1.70 0.40

688 C 689 HN -1.50 0.40

689 C 690 HN 3.70 0.40

690 C 691 HN -3.70 0.40

691 C 692 HN -4.60 0.40

692 C 693 HN 4.50 0.40

693 C 694 HN -2.40 0.40

694 C 695 HN -2.40 0.40

695 C 696 HN -3.50 0.40

696 C 697 HN 4.90 0.40

697 C 698 HN -3.00 0.40

698 C 699 HN -3.50 0.40

699 C 700 HN 1.70 0.40

700 C 701 HN -0.10 0.40

701 C 702 HN -1.60 0.40

702 C 703 HN -5.10 0.40

703 C 704 HN 4.80 0.40

704 C 705 HN -2.00 0.40

705 C 706 HN 2.90 0.40

706 C 707 HN 1.30 0.40

-------------------------------
